# Exploring unconventional food plants used by local communities in a rural area of West Java, Indonesia: ethnobotanical assessment, use trends, and potential for improved nutrition

**DOI:** 10.1186/s13002-024-00710-y

**Published:** 2024-07-19

**Authors:** Yen Yen Sally Rahayu, Wawan Sujarwo, Arifin Surya Dwipa Irsyam, Angga Dwiartama, Dian Rosleine

**Affiliations:** 1 Tokyo College, The University of Tokyo, 7-3-1 Hongo, Bunkyo Ward, Tokyo, 113-8657 Japan; 2 Research Center for Ecology and Ethnobiology, National Research and Innovation Agency (BRIN), Cibinong, Bogor, 16911 Indonesia; 3 School of Life Sciences and Technology, Institut Teknologi Bandung, Jl. Ganesha 10, Bandung, 40132 Indonesia

**Keywords:** Ethnobotany, Underutilized resources, Unconventional food plants, West Java, Consumption

## Abstract

**Background:**

As one of the world’s biodiversity hotspots, Indonesia contains over 25,000 plant species, including unconventional food plants (UFPs). These plants are integral to the dietary practices of rural communities, providing essential nutrients often overlooked in modern diets. However, the use of UFP is declining, with both their dietary and cultural values being undermined. In rural West Java, this decline in UFP biodiversity coincides with public health challenges related to malnutrition. This study aims to document the diversity of UFPs used by local communities in rural West Java, assess their nutritional value, and explore their consumption practices.

**Methods:**

Data were collected using mixed methods, including interviews with 20 key informants and food frequency questionnaire administered to 107 women in three villages in the area. The nutritional compositions of documented UFPs were obtained from literature and analysis. Bivariate correlation was used to analyze the relationships between UFP consumption frequency and potential correlates.

**Results:**

The study documented 52 species of UFPs from 29 families, many of which are rich in nutritional value. About half of respondents (56%) consumed UFPs moderately (2–3 times a week). UFP consumption frequency had a strong correlation (*r* = 0.70) with associated knowledge (*r* = 0.70, *p* < 0.01) and a weak correlation with age (*r* = 0.240, *p* = 0.015), livestock possession (*r* = 0.260, *p* = 0.008), and family size (*r* = − 0.220, *p* = 0.02). Motivations for UFP consumption included availability as free food (33%), medicinal value (26%), nostalgic value (23%), and preferred taste (18%). Most respondents (92%) agreed that consumption has declined compared to the past, with perceived reduced availability and lack of knowledge cited as the primary reasons for the declining trend.

**Conclusions:**

UFP use is common in the study area, where local communities value these plants for their critical roles in diet, medicine, and culture. Given their significant potential to meet dietary needs, educating and raising awareness about UFPs can enhance their consumption and contribute to food and nutrition security.

**Supplementary Information:**

The online version contains supplementary material available at 10.1186/s13002-024-00710-y.

## Introduction

Plants have been essential to human existence, driven by practical needs and cultural traditions [1]. Numerous plant species, particularly food plants, are widely distributed and have well-known applications. Food plants include those with one or more parts that can be used as human food [2], covering plants that are directly consumed, as well as those used to produce oils, spices, and condiments [3–6]. It is estimated that approximately 10% of any given flora represents potential food resources, which implies that of the 270,000 plant species recognized globally, about 27,000 could be considered edible [7]. However, current data show that only 103 species are responsible for 90% of the global food supply, which likely represents an underestimation of the true number of edible plant species [8]. This estimate overlooks many plants with limited distributions, whose uses are either localized or have become neglected [9, 10]. This group of underutilized plants is gaining increasing attention, particularly in response to the expansion of monoculture, and is referred to by various terms such as “famine foods,” “alternative food plants,” “wild edible plants,” “unconventional vegetables,” “traditional vegetables,” and “plants for the future” [2, 10]. These terms often only consider one category of plants (e.g., vegetables, wild, native), which can create ambiguities and require clarification. Thus, it has been proposed to use the term “unconventional food plants” (UFP) to refer to food plants with one or more parts with food potential that are not commonly used [2]. This term also refers to plants that usually do not have market value or are only commercialized on a small scale [2]. Under this broad definition, UFP encompasses native and exotic plants, as well as those that are wild, semi-wild, and cultivated. Even though today’s societies rely mostly on improved varieties, the habit of consuming these underutilized resources has not been entirely abandoned [3]. UFPs have historically been an integral part of dietary practices of rural populations, where these plants are a crucial component of their traditional food systems [10–14].

Despite the prevailing notion that the current global food system provides sufficient calories, approximately two billion people still experience starvation or lack access to a nutritious diet [15]. This issue is further complicated by the phenomenon known as the double burden of malnutrition, which refers to the coexistence of undernutrition and overnutrition and is particularly prevalent in middle-income countries such as many Southeast Asian nations, including Indonesia, Thailand, and Vietnam [16]. Although current food systems generate large volumes of produce, they fall short in supplying sufficient nutrient-dense, plant-based foods essential for healthier and more sustainable diets [10]. As a result, there is an increasing reliance on highly processed, low-nutrient foods, ultimately leading to an overall detrimental nutritional transition [11, 12]. Global diets have increasingly become more uniform, with a 68.8% reduction in the diversity of food supplies across different countries [17]. This trend has led to the predominance of staple crops like wheat, rice, and maize, marginalizing alternative staples that can be considered UFP, such as sorghum, millets, rye, cassava, sweet potato, and yam [10]. Nonetheless, transforming food systems through embracing traditional food practices and exploring nutrient-rich underutilized resources has gained momentum worldwide [11, 18, 19]

With growing recognition of the significance of relocalization and revival of local or traditional food to improve food security and nutrition, UFPs have emerged as a promising avenue [9–11, 18]. These underutilized food resources provide opportunities for diversifying diets with nutrient-rich resources [9, 10]. Despite being under-researched, these UFPs often have better nutritional content than the crops currently dominating our food systems [20]. It was widely reported that traditional varieties often contain higher levels of micronutrients, offering the potential to alleviate micronutrient deficiencies, especially in remote and resource-constrained areas [13, 14]. Their notable nutrient profiles and rich sources of minerals, fiber, vitamins, and fatty acids, making these underutilized resources valuable complements to staple foods [9, 10, 21]. Evidence from Asia indicates that some UFPs that include indigenous vegetables can supplement the daily diet and be used as substitutes for commonly consumed vegetables [22, 23]. Moreover, not only do they play essential roles in diet, but some also offer significant health benefits with well-documented biological and pharmacological effects [4, 23–25].

After Brazil, Indonesia is recognized as the second most biodiverse country globally boasting over 25,000–30,000 plant species [26, 27], out of which 6000 have been harnessed for their applications in food, medicine, and construction materials [28]. As an archipelagic country with over 17,000 islands, Indonesia exhibits significant variations in its culinary traditions and dietary practices, attributed to its diverse geographical, socioeconomic, and cultural characteristics. In this diverse landscape, it has been documented that the Indonesian population has consumed at least 900 edible plants [29]. Previous Indonesian studies have recognized the importance of UFPs in addressing the dietary needs of rural communities. For instance, an agronomy study on indigenous vegetables in the Priangan area of West Java underscored this significance [30]. Additionally, a survey conducted among 157 sellers in East Jakarta traditional markets recorded the sale of 140 edible plants [31]. Ethnobotanical surveys among local people documented 110 native edible plants in the Hulu District of West Kalimantan [32] and 86 species in Bali [33], while another survey conducted on the slopes of Merapi and Merbabu reported the local use of 74 plant species as food [34]. Other ethnobotanical studies have recorded the use of wild edible fruits, documenting a total of 46 species in East Aceh [35], and 73 species in Bengkulu, where these plants were utilized for various other purposes as well [36]. An ethnobotanical survey in the Mentawai Islands of West Sumatra documented the indigenous knowledge and uses of various flora elements and explored their potential role in biodiversity conservation [37]. Additionally, in another region of West Sumatra, a comprehensive study documented 85 species of wild food plants utilized by the Minangkabau and Mandailing people. The study also found that both communities perceive these plants positively [18].

The West Java province is surrounded by mountains, contributing to its high fertility and abundant growth of various plant species [38]. Ethnobotanical surveys conducted in West Java have revealed the extensive utilization of UFPs by the Sundanese people, highlighting their significant role in nutrition, food security, and income generation [28, 38, 39]. Despite their abundance and potential benefits, UFPs have not garnered as much attention as domesticated plants, specifically vegetables. Although most studies have primarily focused on the medicinal value, researchers increasingly recognize these underutilized resources’ significance as an essential alternative source to address the needs of rural populations [4, 24, 40, 41]. On the other hand, the region still faces issues of malnutrition and stunting among children [42, 43], along with an increase in diet-related illnesses rooted in malnutrition [42]. This provincial struggle reflects a broader national issue, where about half of the Indonesian population suffers from at least one micronutrient deficiency, and one in three adults is overweight or obese [16]. Therefore, with its high nutrient content, UFP species can contribute significantly to improving the nutritional quality of the population. However, certain UFP knowledge is confined to particular communities, and because of its vulnerability, such knowledge diminishes rapidly [21]. In recent decades, West Java has been going through major social transformations, and urbanization, which has caused the concentration of population in large cities, has led to the gradual disappearance of traditional food practices and associated knowledge, including UFP use. Moreover, the reliance on these resources is likely to diminish over time due to many factors, among them were the government’s push for commercialization and the promotion of high-yielding cultivars [3], easy accessibility of improved varieties [3, 44], and the decline in species diversity owing to habitat destruction through deforestation, and infrastructure development [18, 45].

Given the challenges and transformations affecting local food practices and biodiversity in West Java, documenting and evaluating traditional knowledge of unconventional food resources is crucial. This ethnobotanical knowledge is primarily preserved within rural communities that maintain traditional practices. This study specifically focuses on Sundanese communities residing in rural West Java to document unconventional food plants (UFPs), assess their nutritional value, and explore their consumption practices. The specific aims are to

1. Document local knowledge of UFPs used in their dietary practice,
2. Assess their nutritional composition and potential contribution to dietary needs,
3. Evaluate the frequency of UFP consumption, and
4. Identify factors associated with UFP consumption and motivations driving their consumption.

## Materials and methods

### Study area and design

**Fig. 1:**
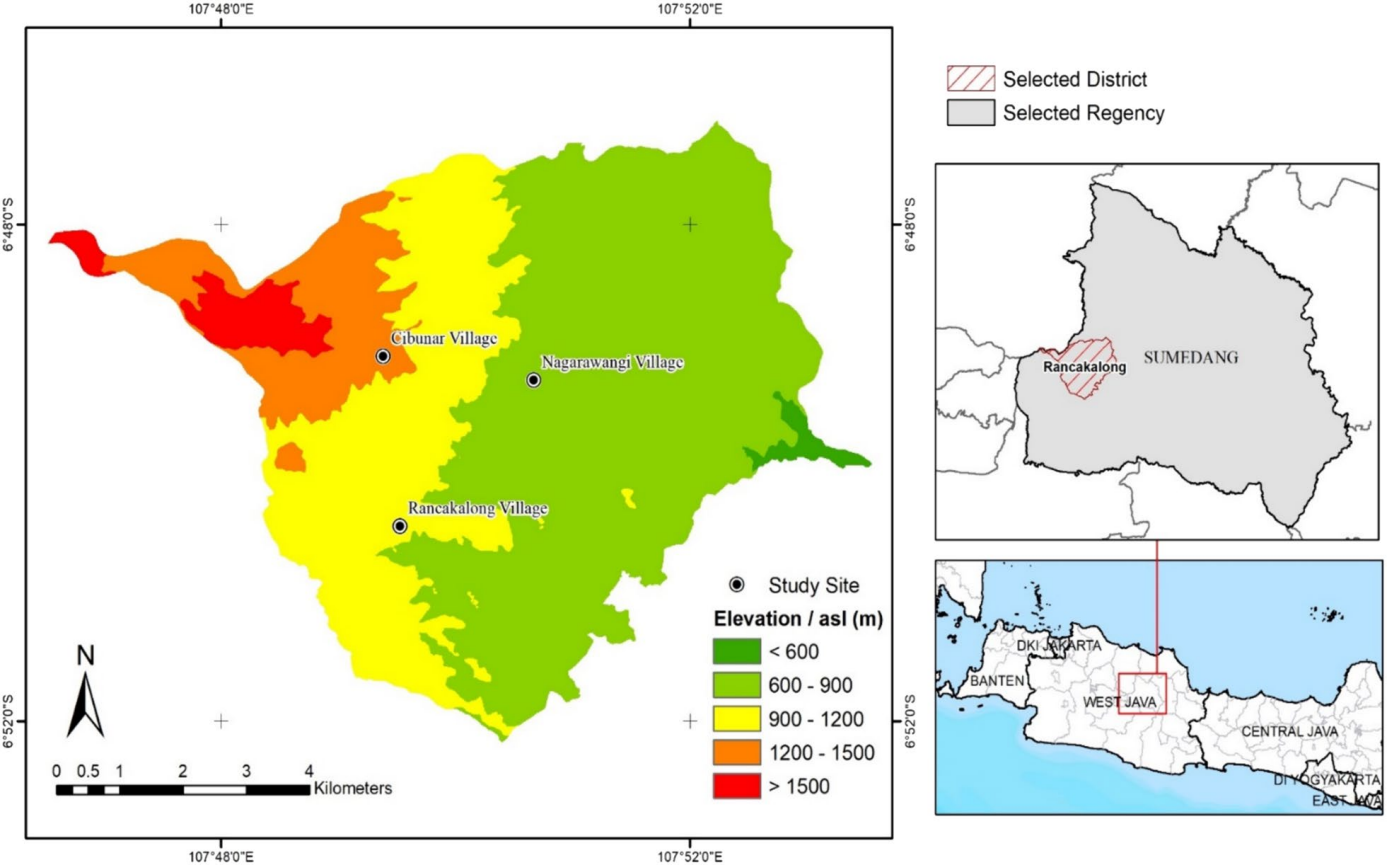
Map of the study area

The data presented in this article were based on surveys undertaken in West Java. The study area, inhabited by Sundanese people, is located in the Rancakalong district of Sumedang Regency, approximately 46 km northeast of Bandung City, the capital of West Java Province (Fig. 1). The local communities in Rancakalong District, the focus of this study, are known for their strong adherence to traditional cultural practices despite the influences of modernization and Islamic traditions. Within Sumedang Regency, which is the epicenter of Sundanese culture in West Java, Rancakalong District is particularly noted as a central cultural hub. Historical traditions are deeply embedded in daily life, especially in agriculture and food practices. Notably, a few older farmers continue to rely on the traditional ecological calendar, known as *pranata wangsa*, to guide their rice cultivation practices. The Rancakalong people highly value the interaction between humans, nature, and God, placing particular importance on expressing gratitude through various festive rituals. One of the most well-known of these rituals is the *ngalaksa*, which is performed as form of gratitude to the rice goddess *Nyi Pohaci* for the harvest. This ritual involves the local communities preparing offerings and various traditional dishes, as well as celebrating with traditional music and dance—Tarawangsa. In Rancakalong, the use of local or traditional food plants is an important aspect of special dishes such cultural ceremonies and ritual offerings, with each plant holding specific symbolic meaning [46].

This study is part of a broader project aiming to enhance diet and nutrition quality by promoting the greater use of local plant biodiversity in rural areas. The area was selected to address this goal as it represents one of the districts in West Java with untapped potential in food plant diversity [47, 48] and continue to uphold traditional practice in various aspects of life, yet it still face persistent malnutrition [49]. Our recent nutritional assessment survey on the site confirms this official report; we found indications of a deficiency in almost all micronutrients (except for sodium) among women (Supplementary 1). The district, a major producer of sweet potatoes, relies predominantly on agriculture as the primary occupation of its residents [50].

We applied a two-phase mixed-methods approach [43] from August 2020 to June 2023, focusing on three villages in the selected district, chosen for their accessibility and the presence of local informants. The names, altitude, and number of houses of each foothill and mountain/migratory village are given in Table 1.

**Table 1:** Profile of study area

	Total area (km^2^)	Number of households	Elevation (m.a.s.l.)	Land use for agriculture and forest cover			
				Wetland paddy field (*sawah*) (%)	Dryland farm (*kebun tegal*) (%)	Community forest (%)	State forest (%)
*District*							
Rancakalong (total 10 villages)	53.6	15,962	835.3 (Average)	25	34	8	20
*Villages*							
Cibunar	4.1	1142	1200–1500 (slope/hilly)	18	26	7	44
Nagarawangi	4.36	2113	600–900 (plain)	24	34	8	22
Rancakalong	3.85	1886	900–1200 (slope/hilly)	21	28	9	34

The first phase employed an ethnobotany survey based on interviews with selected key informants. This survey documented available UFPs in the area and informed the design of questionnaires used in the second phase which was a cross-sectional survey consisting of three modules of semi-structured questionnaires: (1) sociodemographic characteristics; (2) a food frequency for assessing UFPs consumption; and (3) motivation for UFP consumption, and perceptions regarding UFP consumption. The methodology was reviewed by the ethical committee of the Ministry of Health, Bandung Health Polytechnic, Indonesia, and ethical clearance was obtained (No. 05/KEPK/EC/V/2023). The survey was developed to accommodate multiple study objectives and included additional modules not addressed here. The survey materials used in this study are provided upon request.

### Ethnobotany survey and plant identification

Standard ethnobotanical research methods were followed to document local knowledge regarding UFPs through field observations and semi-structured interviews with key informants [51]. Using purposive sampling and the snowball technique, 20 key informants who possess sound traditional knowledge of useful UFPs in the area were selected and interviewed. Therefore, it is important to note that our results may not be interpreted as representative of the whole community. We used this UFP list from these key informants in the Food Frequency Questionnaires (FFQ) administered to respondents of the cross-sectional survey (phase 2): 107 women in the area, with ages ranged from 17 to 80 years with an average of 47.1 ± 13.3 years (Table 2). In the FFQ, we assessed respondents’ knowledge and consumption frequency of listed UFP. We also asked respondents to write down if they knew of any plants that were not on the list. The research was conducted in compliance with the Code of Ethics of the International Society of Ethnobiology Code of Ethics guidelines (ISE 2008). Before starting the interview, the nature of the research and its purpose were explained to obtain verbal consent from all key informants. The interviews were conducted in Indonesian and the traditional Sundanese language and subsequently transcribed into English by the first author, who is proficient in both languages and also a native speaker of Sundanese. We explained to the key informants our intention to gain insights into their knowledge and opinions on the commonly collected and consumed UFPs. Then, the free listing technique was employed to elicit responses from the informants regarding their knowledge of plants and the certain plant parts they used. Each record consisted of details such as the local name of the plant, the specific part that was utilized, and the informant’s perspective on the availability of the species.

**Table 2:** Sociodemographic characteristics of respondents and their UFP use practices (n = 107)

Characteristics	Number	Percentage
*Communities*		
Cibunar village	40	37
Nagarawangi village	35	33
Rancakalong village	32	30
*Ethnicity and language*		
Sundanese	107	100
Bilingualism	103	96
*Religion*		
Islam	100	100
*Average age*	47 ± 13	
*Average household members*	3 ± 1	
*Education completed*		
None	4	4
Primary	73	68
Secondary	15	14
Post Secondary	15	14
*Source of income*		
Farmer/Agriculture-related	54	50
Non-farmer/non-agriculture	53	50
Local shop owners/ food sellers	15	28
Food and handcraft makers	9	17
Laborers	10	19
Homemakers	12	23
Employees	7	13
*Monthly expenditure (in k IDR)*	1677 ± 1172	
*Average number of livestock*	1 ± 0.7	
*UFP-related variables*		
UFP knowledge ^⁎^	6 ± 4	
UFP consumption frequency		
Low	14	13
Moderate	61	56
High	32	30

^*^Average number of UFP recognized by each respondent

Subsequently, the collected data were assessed to verify that the listed plants meet the criteria of UFP in this study. Plants with one or more edible parts that have food potential but are not commonly used or sold and are underutilized in the area, regardless of their status (native or exotic, and wild, semi-wild, semi-cultivated or cultivated), were included as Unconventional Food Plants (UFPs) in this study. Within this criterion, for example, while *Anacardium occidentale* L. is a cultivated crop whose fruits (including nuts) are commonly used and found in the market, its young leaves are considered an uncommon and underutilized food resource and were thus included in the UFP list. Similarly, *Ipomoea batatas* (L.) Lam*.* and *Manihot esculenta* Crantz, which are typically cultivated for their tubers, and *Sicyos edulis* Jacq., cultivated for its fruits, were included because the other edible parts of these crops (leaves, inner peel, and aerial parts, respectively) are considered unconventional and underutilized by the locals. We also included domesticated plants, such as *Averrhoa bilimbi* L.,* Sorghum bicolor* (L.) Moench, which may be commonly used elsewhere [52] but are perceived as neglected or underutilized in the study area. This assessment process resulted in a refined list consisting of 52 UFP species. Plant specimens were collected and subsequently identified by the taxonomist of the School of Life Sciences and Technology, Institut Teknologi Bandung (SITH ITB), who are co-authors of this paper (ASDI) at the Herbarium Bandungense (FIPIA) in West Java. The plants were identified by comparing their characteristics with the literature obtained from reputable sources such as the Flora Malesiana and Flora of China, which are available online at [53–55] as well as Flora of Java [56–58]. The scientific nomenclature was updated based on the World Flora Online Plant List [26].

### Plant nutritional composition data

The nutritional composition of UFPs was obtained from various references and databases, such as the Indonesian Food Composition Table (*Tabel Komposisi Pangan Indonesia*) [59], the Malaysian Food Composition Database (myfcd.moh.gov.my), and the USDA Food Data Central (fdc.nal.usda.gov). Due to the unavailability of information, the composition of *Apoballis rupestris* (Zoll. & Moritzi) S.Y.Wong & P.C.Boyce was evaluated through laboratory analysis conducted at the Test Service Laboratory, Faculty of Agricultural Industrial Technology, Padjajaran University. The study utilized the recommended dietary allowances (RDA) for Indonesia (*Angka Kecukupan Gizi/AKG*) [60] to assess whether the daily intake of a specific plant part was sufficient to meet the recommended nutrient levels. The RDA used in this study was derived from the recommended values for individuals aged 30–49, taking gender differences into account. A plant part was deemed to be a ‘source of a specific nutrient’ if a 100-g portion provided more than 15% of the RDA, and it was considered to have a ‘high content’ if the same 100-g portion contributed more than 30% [61].

### Cross-sectional survey

The cross-sectional survey was conducted to gather information related to UFP use practices. The survey involved participation of 107 women selected through stratified random sampling. Given the overarching goal of the project, which aims to improve diet and nutrition, the survey focused exclusively on women, who typically take on the responsibility of cooking and food preparation in households. This approach is practical for assessing UFPs that were consumed and incorporated into household meals. Moreover, our sampling targeted women aged 15 and older, as this demographic group is known to be vulnerable to malnutrition [62]. The characteristics of the respondents, including attributes related to their UFP practices, are given in Table 2.

Prior to survey administration, informed consent was sought and obtained in written form from all respondents by clearly briefing them about the research objectives, methods, and expected results. During the survey, individual interviews using semi-structured questionnaires were administered to gather information on UFP consumption frequency, its potential correlates (sociodemographic characteristics and UFP-related knowledge), motivation, and perception of the drivers of change in its consumption). The details of these variables are described below.

#### UFP consumption and its potential correlates

UFP consumption was obtained from the Food Frequency Questionnaire (FFQ). The frequency of UFP consumption was classified into four levels: ‘every day’, ‘4–6 times a week’, ‘2–3 times a week’, and ‘once a week or less’ [63]. We assessed sociodemographic variables (i.e., age, number of family members, education level, source of income, monthly expenditure (IDR), and livestock inventory), and UFP-related knowledge as potential correlates of UFP consumption.

The sociodemographic variables are respondents’ self-reported values. UFP knowledge was assessed based on the number of species that could be recognized on the key informants' list.

#### Motivation and perception of the drivers of change in UFP consumption

Motivation and perception on UFP consumption were collected through open-ended questions. A thematic analysis was used to identify emerging themes from respondents’ comments regarding the motivation for UFP consumption, perceived consumption trend compared to the past, and factors driving this change. We compared the reasons for consuming UFP with those of commonly consumed vegetables.

### Statistical analysis

SPSS software was used for data entry and analysis to provide measures of frequency and correlation between variables. We applied Pearson’s and Spearman’s correlations to explore the relationships between UFP consumption frequency and its potential correlates. Pearson’s correlation was used for parametric variables that are normally distributed, while Spearman’s was used for non-parametric variables or when the data from one or both variables do not follow normal distributions [64]. The Kolmogorov–Smirnov and Shapiro–Wilk tests were employed to examine data distribution. In the analysis of UFP consumption frequency, ‘every day’ and ‘4–6 times a week’ are categorized as high consumption, ‘2–3 times a week’ as moderate consumption, and ‘once or less’ as low consumption. The source of income is input as a dummy variable (non-farme* r* = 0, farme* r* = 1), and the ordinal variable on education completed is treated as continuous (none = 0, elementary school = 1, junior high school = 2, high school = 3, higher than HS = 4).

## Results and discussion

### Diversity of UFP species

This study recorded a total of 52 species, which were classified into 29 families (Table 3). This number surpasses documented in other ethnobotany studies on unconventional food resources in the Southeast Asia region, such as those documenting indigenous vegetables and wild food plants used by indigenous communities of northern Thailand [65], Southern Shan State in Myanmar [66], Lao PDR [67], and the Philippines [68]. However, it is fewer compared to a study conducted in three regencies in the eastern part of West Java—namely Tasikmalaya, Ciamis, and Garut—which reported 86 species of indigenous vegetables [38]. Similarly, studies in other regions of Indonesia recorded 85 wild food plants in the Pasaman regency of West Sumatera [18], while [38] identified 96 species found near a forest area in Kapuas Hulu regency in West Kalimantan. These differences may be attributed to differences in the emic categorization of species considered in the interviews and the smaller coverage in our study. The fewer documented edible plants in our study can also support the declining availability perceived by respondents, the details of which will be deferred later. The number of UFPs documented in the present study is comparable to that reported in the Mekong Delta in Vietnam [69], tropical rain forests in Sarawak of Malaysia [70], and in other areas across Asia, such as Pakistan [21], Eastern Bhutan [3], Western Himalaya [71], Far west Nepal [72] and Ethiopian regions [73, 74]. Moreover, the discrepancies in the number of plants recorded can also be explained by the use of *ex situ* methods—free listing and FFQ (dietary recall) survey—for plant documentation. *Ex situ* methods tend to gather less information about the plants and their usage compared to the in situ “walk-in-the-woods” method [75].

**Table 3:** List of unconventional food plants used by local communities in the study area

Family	Species name	Collection number	Local (Sundanese) name	Edible part	Consumption mode	Other food ethnobotanical reports in Indonesia
Achariaceae	*Pangium edule* Reinw.	AC01/N	Picung	Kernel/seeds	Seeds are soaked for a few days before cooking to remove toxins from the fruit. Afterward, the seeds can be opened to reveal a white to yellowish flesh called *dage*, the part used for cooking. It is commonly used in coconut milk-based dishes like *sayur lodeh* or simply sautéed with spices	[32, 33, 159, 160]
Altingiaceae	*Liquidambar excelsa* (Noronha) Oken	MR08/N	Rasamala	Young leaves	Raw or boiled as *lalapan*	[161, 162]
Apiaceae	*Centella asiatica* (L.) Urb.	RM42/R	Antanan	Leaves	Edible parts are consumed raw as *lalapan* or used in *rujak*	[30, 38, 79–81]
	*Eryngium foetidum* L.	AC02/N	Welang, katuncar walanda	Leaves	Boiled as *lalapan* or for used in noodle soup	[32, 38, 80]
	*Oenanthe javanica* DC.	AC03/N	Tespong	Aerial parts	Raw as *lalapan*	[38, 80, 82]
Anacardiaceae	*Anacardium occidentale* L.	AC04/N	Jambu mede	Young leaves	Raw as lalapan,	[30, 39, 80, 163]
	*Mangifera foetida* Lour.	AC05/N	Limus	Fruits	Raw when ripe, and unripe ones are used in *rujak* or *manisan* (preserved fruits in syrup)	[28, 36, 164, 165]
	*Mangifera odorata* Griff.	AC06/N	Kaweni	Fruits	Raw when ripe, and unripe ones are used in *rujak* or *manisan* (preserved fruits in syrup)	[28, 35, 36, 166]
Araceae	*Apoballis rupestris* (Zoll. & Moritzi ex Zoll.) S.Y.Wong & P.C.Boyce	AY01/C	Cariwuh	Rhizomes	Rhizomes are steamed or cooked with other ingredients to make a traditional dish called *pahinum*	–
Asteraceae	*Acmella ciliata* (Kunth) Cass.	RM01/S	Jotang	Aerial parts	Raw as *lalapan*	[160, 167]
	*Cosmos caudatus* Kunth	RM02/S	Randamidang	Leaves	Raw or boiled as *lalapan* or used in a mixed (steamed) vegetable dish called *urab*	[30, 82]
	*Crassocephalum crepidioides* S.Moore	RM/08/R	Sintrong	Leaves	Raw as *lalapan*	[38, 84, 85]
	*Gynura divaricata* DC.	RM05/R	Dewa	Leaves	Raw as *lalapan*	[38]
	*Erigeron sumatrensis* Retz.	RM06/S	Jalantir	Leaves	Boiled as *lalapan*	[85]
	*Bidens pilosa* L.	RM04/S	Hareuga	Aerial parts	Raw or boiled as *lalapan*	[167]
	*Emilia sonchifolia* (L.) DC.	AY05/C	Jonge, jonghe, jeletun	Leaves	Raw or boiled as *lalapan*	[38, 80, 167]
	*Sonchus arvensis* L.	MR03/N	Lampenas, camawak	Leaves	Raw or boiled as *lalapan*	[82]
Athyriaceae	*Diplazium esculentum* (Retz.) Sw.	RM08/S	Pakis	Aerial parts	Boiled and cooked as a sautéed dish	[33, 80, 165, 168, 169]
Brassicaceae	*Rorippa indica* (L.) Hiern	MR05/N	Kamanilaan	Leaves	Raw or boiled as *lalapan*	[79]
Cannaceae	*Canna indica* L.	RM09/S	Ganyol	Rhizomes	Cooked (boiled/steamed/roasted/fried) as a snack	[170]
Convolvulaceae	*Ipomoea batatas* (L.) Lam.	RM10/S	Hui	Young leaves	Cooked as a sautéed dish	[38, 168]
Cucurbitaceae	*Benincasa hispida* Cogn.	RM11/S	Baligo	Fruits	Cooked in various kinds of soups	[79]
	*Lagenaria siceraria* (Molina) Standl.	RM12/S	Anolekak, kukuk	Fruits	Cooked in various kinds of soups, including coconut milk-based dishes	[38]
	*Sicyos edulis* Jacq.	AC07/N	Waluh	Leaves and stems	Boiled for *lalapan* or used in a mixed vegetable dish called *lotek* (peanut sauce salad)	[30, 39, 79, 160]
Euphorbiaceae	*Manihot esculenta* Crantz	RM62/R	Sampeu	Inner peel/ cortex	Boild and cooked with spices	[38, 79, 80, 84, 160]
Fabaceae	*Archidendron jiringa* (Jack) I.C.Nielsen	AY02/C	Jengkol	Seeds	For *lalapan,* immature seeds are eaten raw, while ripe ones are fried. Ripe seeds can also be stewed with coconut milk	[79, 137, 163, 166]
	*Clitoria ternatea* L.	RM13/S	Telang	Flowers	Dried to be used as tea	[34, 81, 171]
	*Leucaena leucocephala* (Lam.) de Wit	RM04/R	Selong, peuteuy selong	Fruits	Raw or boiled as *lalapan*	[31, 34, 79, 80, 172]
Hydrocharitaceae	*Limnocharis flava* (L.) Buchenau	AC08/N	Eceng, genjer	Aerial parts	Raw or boiled as *lalapan*, cooked as a sautéed dish	[30, 80, 160, 165, 173]
Malvaceae	*Durio zibethinus* L.	RM15/S	Kadu	Fruits	Raw on ripe	[28, 34, 79, 84, 159, 160, 164, 170, 173]
Melastomataceae	*Melastoma malabathricum* L.	RM16/S	Harendong	Fruits	Raw on ripe	[36, 79, 85, 159, 165, 170]
Meliaceae	*Sandoricum koetjape* Merr.	RM17/S	Kacapi	Fruits	Raw on ripe	[28, 35, 168]
Menispermaceae	*Cyclea barbata* Miers	RM67/S	Cincau	Leaves	Cooked to make jelly	[79, 165, 174]
Moraceae	*Artocarpus altilis* (Parkinson) Fosberg	AY03/C	Kelewih*	Fruits	Fried as a snack, or cooked as a sautéed or stewed dish	[34, 83, 163, 166, 172]
	*Ficus virens* Aiton	MR01/N	Bunut	Young leaves	Raw as *lalapan*	[36, 175, 176]
Moringaceae	*Moringa oleifera* Lam.	RM28/R	Kelor	Leaves	Leaves are cooked, usually sautéed with various spices	[35, 38, 81]
Muntingiaceae	*Muntingia calabura* L.	RM21/S	Kersen	Fruits	Raw on ripe	[28, 79]
Myrtaceae	*Syzygium cumini* (L.) Skeels	RM22/S	Duwet	Fruits	Raw on ripe	[28, 35, 79, 175, 176]
Oxalidaceae	*Averrhoa bilimbi* L.	RM17/R	Calincing	Fruits	Raw as a snack or used in *rujak*	[28, 31, 33, 160, 170, 173]
Phyllanthaceae	*Antidesma bunius* (L.) Spreng.	RM24/S	Huni	Fruits	Raw as a snack or used in *rujak*	[28, 168, 174]
	*Baccaurea racemosa* (Reinw.) Müll.Arg.	RM25/S	Bencoy	Fruits	Raw as a snack or used in *rujak*	[28, 36, 79, 160, 165, 175]
	*Breynia androgyna* (L.) Chakrab. & N.P. Balakr.	RM 26/R	Katuk	Leaves	Cooked as sauted dish	[79, 82, 165]
	*Phyllanthus acidus* (L.) Skeels	AY07/C	Cereme	Fruits	Raw as a snack or used in *rujak*	[28]
	*Phyllanthus emblica* L.	RM26/S	Ki malaka	Fruits	Raw as a snack or used in *rujak*	[28, 170]
Piperaceae	*Piper retrofractum* Vahl	AC11/N	Cabe jawa	Fruits	Used raw in seasoning	[81, 170]
Poaceae	*Sorghum bicolor* (L.) Moench	RM/44R	Gandrung	Grains	Cook similarly to rice, or pounded into flour and used for making various desserts	[77]
Solanaceae	*Physalis angulata* L.	RM29/S	Cecenet	Fruits	Raw on ripe	[35, 36, 81, 84, 85, 160, 173]
	*Solanum americanum* Mill.	RM30/S	Leunca	Fruits	Raw as *lalapan* or cooked as sauted dish with *oncom* (*fermented tempeh*)	[30, 39, 137]
	*Solanum torvum* Sw.	AY04/C	Takokak	Fruits	Raw or boiled as *lalapan,* cooked as sauted dish	[38, 79, 80, 137, 159, 160, 170]
Urticaceae	*Pilea melastomoides* (Poir.) Wedd.	AC09/N	Poh pohan	Leaves	Raw as *lalapan*	[38, 82, 161, 165, 167]
Zingiberaceae	*Amomum dealbatum* Roxb.	RM32/S	Rangasa, hanggasa	Fruits	Raw on ripe or candied	[52, 177]
	*Etlingera elatior* (Jack) R.M.Sm.	RM33/S	Honje	Flowers	Raw as *lalapan*, or used in a salad mixture or as a flavoring in dishes	[31, 32, 38, 160, 161]

*Non-seeded type (sterile)

*Lalapan*—also known as lalab or lalap, is a popular Sundanese vegetable dish originating from West Java, Indonesia. It refers to raw or boiled vegetables and is usually served with sambal, a spicy traditional dip or thick sauce. While it is comparable to a vegetable salad in Western culture, a pair of lalapan and sambal is considered a main course

*Rujak*—is a sweet and spicy dressing or dip made from thick palm sugar syrup, shrimp paste (*terasi*), and chili, typically used in Indonesian fruit salads. The term ‘rujak’ also commonly refers to the fruit salad itself, which is served with this distinctive dressing or dip

According to our literature review listed in Table 3, all the species documented in this study have been previously reported by food ethnobotanical research in Indonesia, except for *Apoballis rupestris*. *Apoballis rupestris*—native to Java, the Lesser Sunda Islands, and Sumatra—was previously reported in ethnobotany studies as an ornamental plant [76, 77]. This present study is the first to report this species in the context of food plants, thus contributing to the knowledge of the diversity of food plants in the country. Furthermore, 30 of 52 documented UFPs were reported in the ethnobotanical inventories for both food and medicinal purposes, including the nationwide ethnomedicine project (RISTOJA) conducted by the Indonesian Health Authority [78]. These include *Centella asiatica* [4, 30, 38, 40, 79–81], *Eryngium foetidum* [32, 38, 40, 80], *Oenanthe javanica* [38, 80, 82], *Cosmos caudatus* [30, 82, 83], *Crassocephalum crepidioides* [4, 38, 84, 85], *Gynura divaricata* [4, 38], and *Bidens pilosa* [38, 85, 86].

Among documented UFPs in this study, the most diverse plant family was Asteraceae (8 species), followed by Phyllanthaceae (5 species). The families Apiaceae, Anacardiaceae, Cucurbitaceae, Fabaceae, and Solanaceae each had 3 species each. Other families consist of two or fewer species each. Asteraceae was consistently reported in different ethnobotanical documentation of edible plants in Java as one of the families with the highest contribution of edible species [38, 81]. Regarding the different plant parts used, the fruits were most prominently consumed (22%), followed by leaves (18%), and aerial parts (leaves, shoots, and stems). Other parts consumed included inner peel, grains, seeds (including kernels), and their flowers. These plant parts are usually gathered and consumed as vegetables, spices/condiments, snacks/delicacies, and fruits. In the present study, the most widely used UFPs were those regularly consumed as vegetables, referred to as *lalapan* in Indonesian. These UFPs include *Solanum americanum*, *Crassocephalum crepidioides, Solanum torvum, Eryngium foetidum, Moringa oleifera*, and *Breynia androgyna*. Local people in the western Himalaya also used uncultivated edible plants to meet their vegetable requirements, as did the Chepang people in Nepal [71]. Furthermore, non-cultivated greens were a major source of vegetables in rural areas of Vietnam [69].

Food plants in West Java, particularly leafy greens, play a crucial role in the Sundanese diet [38]. The Sundanese people are well known for their traditional dietary practice of consuming fresh vegetables, known as *lalab* (Sundanese) or *lalap/lalapan* (Indonesian) [4], in their daily meals, similar to Western salads. Most *lalap*an are traditionally consumed raw and typically served with *sambal*, a spicy paste condiment made from a mixture of chili and other secondary ingredients such as shrimp paste, shallots, palm sugar, and lime juice, comparable to a dressing in salad, a dip or salsa. Unlike salads, which are often served as appetizers, Sundanese *lalapan* served with *sambal* is considered a main dish. Initially associated with the Sundanese food culture, *lalapan* has become indispensable in Indonesian gastronomy and includes all types of vegetables [38]. However, in the specific context of Sundanese communities, including in the study area, *lalapan* encompasses wild, semi-wild, semi-cultivated, and cultivated food plants [38]. The integration of UFPs of local communities’ diet in the present study, particularly in the form of *lalapan*, highlights the importance of these underutilized plants in maintaining traditional dietary practices. The documentation of UFPs in this study highlights the existing diversity of underutilized plants resources in the area. Although there are fewer UFPs than in some other regions, this presents an opportunity to expand the exploration and documentation of local food resources, which could ultimately strengthen the role of UFPs in the local food system.

### Nutritional compositions and potential contributions to recommended dietary allowances (RDA)

The nutritional compositions (protein, fiber, Ca, Fe, Zn, and vitamin C) of the documented UFPs vary widely among the species (Table 4). Note that, due to the limited available references, some nutrient values represent single data points rather than averages. Additionally, following [23, 61], the percentage contributions of each plant’s nutrient content to the recommended dietary allowances (RDA) were calculated based on 100 g of plants, which aligns with the standard serving size for raw vegetables in Indonesian dietary guidelines [87]. Numerous studies underscore the high nutritional value of underutilized food plants [10, 61, 64, 88, 89], as evidenced by different documented groups of UFPs in this study. Their potential contribution to the RDA demonstrates that some of them can serve as a ‘source’ (RDA > 15%) or contain ‘high level’ (RDA > 30%) of certain nutrients (Table 5).

**Table 4:** Composition in macronutrients (protein, fiber), minerals (Ca, Fe, Zn), and vitamin C of some documented UFPs

Species	EP	Protein (g/100 g)	Fiber (mg/100 g)	Ca (mg/100 g)	Fe (mg/100 g)	Zn (mg/100 g)	Vit. C (mg/100 g)	References
*P. edule*	S	8.7 (7.3–10.0)	9.6 (–)	41 (40–42)	2.05 (2.0–2.1)	1.4 (–)	24.5 (19–30)	[59, 70]
*C. asiatica*	L	7.3 (2.7–11.9)	2.2 (1.8–2.7)	177 (–)	4.1 (–)	8.3 (7.5–10.9)	0.7 (–)	[23, 110]
*E. foetidum*	L	1.0 (0.7–1.2)	1.2 (1.2–1.3)	57 (48–67)	4 (1.8–7.2)	0.6 (–)	75 (17–133)	[103, 117, 125, 126]
*O. javanica*	Ap	2.1 (1.2–2.9)	2.0 (1.1–2.9)	152 (133–170)	3.6 (1.4–7.0)	7.1 (0.15–14.0)	12 (3–21)	[23, 59, 116, 117]
*A. occidentale*	Yl	5.3 (3.7–7.0)	2.3 (1.5–3.0)	24 (16–33)	4.7 (0.5–8.9)	–	59 (21–91)	[39, 59, 111, 116]
*M. foetida*	F	1.1 (0.8–1.4)	1.9 (1.8–2.0)	26 (16–36)	0.25 (0.2–0.3)	–	52 (47–56)	[111, 178]
*M. odorata*	F	1.1 (0.7–1.4)	4.2 (–)	15 (9–21)	0.35 (0.2–0.5)	0.1 (–)	37 (18– 56)	[35, 59]
*A. rupestris*	R	4.7 (–)	3.0 (–)	24 (–)	1.03 (–)	0.3 (–)	22 (–)	Experiment
*A. ciliata*	Ap	2.2 (1.9–2.5)	2.6 (1.6–3.5)	117 (71–162)	7.5 (4.0–11)	1.7 (1.2–2.2)	20 (–)	[59, 121]
*C. caudatus*	L	3.5 (2.9–4.2)	3.0 (1.6–5.8)	299 (279–328)	3.7 (2.7–4.6)	0.6 (–)	58 (0–109)	[39, 52, 59, 111]
*C. crepidioides*	L	6.6 (2.6–13.6)	6.8 (1.7–11.9)	183 (17–398)	4.9 (0.5–9.3)	0.4 (0.4–0.5)	38 (3–73)	[59, 104, 112, 113]
*G. divaricata*	L	6.7 (6.4–7.0)	13.8 (6.0–21.5)	491 (–)	5.0 (–)	–	4 (–)	[102, 103]
*E. sumatrensis*	L	17.5 (–)	12.7 (–)	12 (–)	1.7 (–)	–	0.6 (–)	[95, 179]
*B. pilosa*	Ap	3.3 (2.3–4.2)	2.6 (1.3–3.9)	225 (110–340)	8.3 (2.3–14.2)	1.2 (–)	40 (–)	[122, 123]
*E. sonchifolia*	L	1.9 (1.6–2.1)	3.2 (2.0–4.3)	133 (52–253)	6.0 (3.6–9.5)	0.2 (–)	1.5 (1.0–1.9)	[59, 103, 124]
*S. arvensis*	L	1.1 (0.1–0.2)	8.5 (0.3–1.6)	854 (6.3–1702)	6.2 (0.3–12.1)	2.3 (0.02–4.65)	64 (–)	[106, 107]
*D. esculentum*	L	4.0 (3.4–4.5)	3.2 (2.0–4.3)	75 (13–136)	1.8 (1.3–2.3)	0.5 (0.3–0.7)	6.5 (3–10)	[59]
*C. indica*	R	0.8 (0.6–1.0)	0.8 (–)	18 (15–21)	10.5 (1.0–20.0)	–	9.5 (9–10)	[59, 120]
*I. batatas*	Yl	3.6 (3.0–4.1)	7.0 (6.4–7.6)	157 (80–258)	3.5 (0.6–6.4)	0.4 (0.4–0.5)	11.5 (4–27)	[59, 105]
*B. hispida*	F	0.5 (0.3–0.7)	1.1 (0.5–1.7)	14 (5–23)	0.3 (0.2–0.5)	0.2 (–)	35 (1.4–69)	[130, 131]
*L. siceraria*	F	0.6 (0.6–0.62)	0.6 (0.5–0.6)	19 (12–25)	4.1 (0.2–7.9)	0.7 (–)	49 (10–88)	[59, 180, 181]
*S. edulis*	Ls	3.6 (3.6–4.0)	3.2 (1.1–21.7)	98 (58–138)	3.1 (2.5–3.7)	0.3 (–)	26.0 (16–36)	[59]
*M. esculenta*	Ip	0.02 (–)	12.4 (–)	–	–	–	–	[90, 91]
*A. jiringa*	S	5.4 (–)	1.5 (–)	4 (–)	0.7 (–)	0.6 (–)	31 (–)	[59]
*C. ternatea*	Fl	0.02 (–)	0.2 (–)	8 (–)	1.1 (–)	4.5 (–)	–	[127]
*L. leucocephala*	S	18.4 (5.7–31)	13.6 (10.8–16.4)	490 (180–800)	1.9 (1.2–2.7)	3.6 (1.4–5.8)	15 (–)	[59, 92, 93]
*L. flava*	Ap	1.4 (1.0–1.7)	2.2 (1.8–2.5)	71 (62–80)	2.9 (2.1–3.7)	–	52 (50–54)	[59]
*D. zibethinus*	F	2.4 (1.7–3.5)	2.6 (1.2–4.4)	31 (4.5–190)	1.0 (0.4–2.9)	0.4 (0.15–1.40)	42 (23–107)	[59, 182–184]
*M. malabathricum*	F	5.3 (5.1–5.5)	8.6 (–)	152 (2.5–302)	4.3 (0.5–8.0)	–	–	[185, 186]
*S. koetjape*	F	2.3 (0.4–4.1)	13.9 (1.0–26.8)	57 (4–110)	1.7 (1.2–2.1)	–	14 (–)	[35, 99]
*C. barbata*	L	4.2 (–)	9.8 (–)	237 (–)	–	–	–	[35]
*A. altilis*	F	2.7 (0.1–5.2)	0.9 (0.2–1.5)	31 (24–37)	1.5 (1.4–1.6)	0.1 (–)	55 (52–58)	[59, 187]
*M. oleifera*	L	16.3 (5.1–28)	10.2 (1.2–19.3)	593 (261–1077)	5.3 (3.0–7.0)	0.6 (–)	64 (22–106)	[59, 96]
*M. calabura*	F	4.3 (0.3–8.3)	5.3 (4.6–5.9)	124 (–)	1.2 (–)	–	42 (3.3–80.5)	[188–190]
*S. cumini*	F	1.0 (0.5–1.4)	0.6 (0.2–0.9)	31 (0–62)	1.1 (0.1–2.0)	1.2 (0.3–2.1)	31 (5.7–56)	[59, 129]
*A. bilimbi*	F	0.7 (0.6–0.7)	0.6 (0.6–0.7)	7 (3.4–12.0)	1.5 (0.4–3.2)	0.04 (–)	60 (15.5–183)	[111, 191–194]
*A. bunius*	F	0.1 (0.5–0.75)	3.3 (–)	109 (0.1–279)	1.2 (0.1–2.7)	1.0 (–)	38 (7.3–69)	[52, 132, 133]
*B. racemosa*	F	1.7 (–)	–	13 (–)	0.8 (–)	–	–	[59]
*B. androgyna*	L	5.1 (0.8–8.3)	1.0 (0.25–1.5)	152 (12– 313)	5.2 (1.9 –10.1)	1.9 (1.3–2.5)	69 (24–136)	[52, 59, 114, 115]
*P. acidus*	F	0.7 (–)	0.6 (0.5–0.6)	7 (5–9)	0.4 (0.36–0.4)	0.15 (–)	8 (–)	[195–197]
*P. emblica*	F	2.3 (0.7–4.0)	12.5 (3.0–22.0)	95 (0–189)	2.2 (0.2–4.3)	1.0 (0.06–2.0)	65 (33–97)	[100, 101]
*P. retrofractum*	F	9.3 (–)	23.6 (–)	340 (–)	4.2 (–)	0.8 (–)	–	[97]
*S. bicolor*	G	7.6 (4.3–11.0)	1.8 (–)	14 (0–28)	2.2 (0.03–4.4)	0.9 (0.5–1.4)	0	[57, 198, 199]
*P. angulata*	F	7.3 (3.7–11.0)	7.7 (4.4–11.0)	12 (0–24)	3.1 (0.2–6.0)	0.02 (–)	46 (46–47)	[198–201]
*S. americanum*	F	3.6 (1.1–7.9)	5.5 (2.0–14.5)	260 (7–515)	6.3 (1.0–11.7)	0.3 (–)	15 (13.5–17)	[52, 59, 103]
*S. torvum*	F	2.3 (1.6–2.8)	6.6 (3.8–10.3)	160 (50–329)	3.5 (0.6–7.1)	0.7 (0.4–1.0)	15 (4.0–38)	[59, 108, 109]
*P. melastomoides*	L	2.9 (2.5–3.3)	2.6 (–)	744 (–)	5.9 (–)	–	25 (5–45)	[39, 59]
*A. dealbatum*	F	3.1 (–)	6.5 (–)	–	–	–	–	[128]
*E. elatior*	Fl	1.2 (0.9–1.3)	2.2 (1.2–2.8)	57 (32–78)	1.7 (0.2–4.0)	0.4 (0.1–1.0)	0.7 (0–1.4)	[59, 202, 203]

The value represents the mean value with the range of variability in the literature in the parentheses

*EP* edible part, *S* seeds, leaves, *A* aerial parts, *Yl*: young leaves, *F* fruits, *R* rhizomes, *Ls* leaves and stems, *Ip* inner peel/cortex, *Fl* flowers, *G* grains

**Table 5:** Percentage contribution of 100 g edible parts of some documented UFPs to Recommended Dietary Allowances (RDA) for adults (30–49 years) for proteins, fiber, mineral elements (Ca, Fe, Zn), and vitamin C

Plant species	EP	% RDA (male/female)*					
		Protein	Fiber	Ca	Fe	Zn	Vit. C
		65/60	36/30	1000	9/18	11/8	90/75
		g/day	g/day	mg/day	mg/day	mg/day	mg/day
*P. edule*	S	13.3**/**14.4	*26.7*/**32.0**	4	*22.8*/11.4	12.7/*17.5*	*27.2*/**32.7**
*C. asiatica*	L	11.2/12.1	6.2/7.5	*18*	**45.6**/*22.8*	**75.1**/**103.2**	0.8/1.0
*E. foetidum*	L	1.5/1.6	3.5/4.2	6	**49.6**/*24.8*	5.4/7.4	**83.3**/**99.9**
*O. javanica*	A	3.2/3.4	5.5/6.6	*15*	**40.2**/*20.1*	**64.3**/**88.4**	13.5/*16.2*
*A. occidentale*	Yl	8.2/8.9	6.3/7.5	2	**52.4**/*26.2*	–	**65.5**/**78.6**
*M. foetida*	F	1.7/1.8	5.3/6.3	3	2.81.4	–	**57.4**/**68.9**
*M. odorata*	F	1.6/1.8	11.7/14.0	1	3.9/1.9	0.9/1.3	**41.1**/**49.3**
*A. rupestris*	R	7.2/7.8	8.3/10.0	2	11.4/5.7	2.9/4.0	*24.4*/*29.3*
*A. ciliata*	A	3.4/3.7	7.1/8.5	12	**83.3**/**41.7**	*15.5*/*21.3*	*22.2*/*26.7*
*C. caudatus*	L	5.3/5.8	8.3/10.0	*30*	**40.6**/*20.3*	5.5/7.5	**64.2**/**77.1**
*C. crepidioides*	L	10.1/11.0	*18.9*/*22.7*	*18*	**54.5**/*27.3*	4.1/5.6	**42.4**/**50.9**
*G. divaricata*	L	10.3/11.2	**38.3**/**45.9**	**49**	**55.0**/*27.5*	–	4.4/5.3
*E. sumatrensis*	L	*26.8*/*29.1*	**35.2**/**42.2**	1	*18.7*/9.3	–	0.6/0.8
*B. pilosa*	A	5.0/5.4	7.2/8.7	*23*	**91.7**/**45.8**	10.7/14.8	**44.4**/**53.3**
*E. sonchifolia*	L	2.8/3.1	8.8/10.5	13	**66.4**/**33.2**	1.8/2.5	1.6/1.9
*S. arvensis* L	L	1.7/1.8	*23.6*/*28.3*	**85**	**68.7**/**34.3**	*21.2*/*29.2*	**70.7**/**84.9**
*D. esculentum*	L	6.1/6.6	8.8/10.5	7	*20.0*/10.0	4.5/6.3	7.2/8.7
*C. indica*	R	1.2/1.3	2.2/2.7	2	**116.7**/**58.3**	–	10.6/12.7
*I. batatas*	Yl	5.5/6.0	*19.4*/*23.3*	*16*	**38.9**/*19.4*	4.0/5.5	12.8/*15.3*
*B. hispida*	F	0.8/0.8	3.1/3.7	1	3.8/1.9	1.8/2.5	**39.1**/**46.9**
*L. siceraria*	F	0.9/1.0	1.5/1.8	2	**45.0**/*22.5*	6.4/8.8	**54.3**/**65.2**
*S. edulis*	Ls	5.6/6.1	8.9/10.7	10	**34.4**/*17.2*	2.7/3.8	*28.9*/**34.7**
*M. esculenta*	Ip	0.035/0.04	**34.5**/**41.4**	–	–	–	–
*A. jiringa*	S	8.3/9.0	4.2/5.0	0.4	7.8/3.9	5.5/7.5	**34.4**/**41.3**
*C. ternatea*	Fl	0.04/0.04	0.4/0.5	0.8	12.2/6.1	**41.3**/**56.8**	–
*L. leucocephala*	S	*28.3*/**30.7**	**37.8**/**45.4**	**49**	*21.5*/10.8	**32.9**/**45.2**	*16.7*/*20.0*
*L. flava*	A	2.1/2.3	6.0/7.2	7	**32.2**/*16.1*	–	**57.8**/**69.3**
*D. zibethinus*	F	3.7/4.1	7.1/8.5	3	11.5/5.7	3.9/5.4	**46.3**/**41.3**
*M. malabathricum*	F	8.1/8.8	*23.8*/*28.5*	*15*	**47.4**/*23.7*	–	–
*S. koetjape*	F	3.5/3.8	**38.6**/**46.4**	6	*18.3*/9.2	–	*15.6*/*18.7*
*C. barbata*	L	6.5/7.0	*27.2*/**32.7**	*24*	–	–	–
*A. altilis*	F	4.1/4.4	2.4/2.8	3	*16.7*/8.3	0.9/1.3	**61.1**/**73.3**
*M. oleifera*	L	*25.1*/*27.2*	*28.4*/**34.1**	**59**	**59.3**/29.6	5.5/7.5	**71.1**/**85.3**
*M. calabura*	F	6.6/7.2	14.6/*17.6*	12	13.1/6.6	–	**46.6**/**55.9**
*S. cumini*	F	1.5/1.6	1.6/1.9	3	11.7/5.9	10.9/*14.9*	**34.3**/**41.1**
*A. bilimbi*	F	1.0/1.1	1.8/2.1	1	*17.1*/8.6	0.4/0.5	**66.3**/**79.6**
*A. bunius*	F	0.2/0.2	9.3/11.2	11	12.9/6.4	9.3/12.8	**42.4**/**50.9**
*B. racemosa*	F	2.6/2.8	9.3	1	8.9/4.4	–	–
*B. androgyna*	L	7.9/8.5	2.9/3.4	*15*	**57.4**/*28.7*	*17.0*/*23.4*	**76.3**/**91.6**
*P. acidus*	F	1.1/1.2	1.5/1.8	1	4.2/2.1	1.4/1.9	8.9/10.7
*P. emblica*	F	3.6/3.9	**34.7**/**41.7**	9	*24.6*/12.3	9.3/12.9	**72.3**/**86.8**
*P. retrofractum*	F	14.4/*15.6*	**65.6**/**78.7**	**34**	**46.6**/*23.3*	6.9/9.5	–
*S. bicolor*	G	11.7/12.7	5.0/6.0	1	*24.6*/12.3	8.6/11.8	–
*P. angulata*	F	11.2/12.2	*21.4*/*25.6*	1	**34.5**/*17.2*	0.2/0.3	**51.6**/**62.0**
*S. americanum*	F	5.6/6.1	*15.3*/*18.3*	*26*	**70.3**/**35.1**	2.7/3.8	*17.0*/*20.3*
*S. torvum*	F	3.5/3.8	*18.4*/*22.1*	*16*	**39.3**/*19.7*	6.4/8.8	*17.0*/*20.4*
*P. melastomoides*	L	4.5/4.9	7.2/8.7	**74**	**65.6**/**32.8**	–	*27.9*/**33.4**
*A. dealbatum*	F	4.8/5.2	*17.9*/*21.5*	–	–	–	–
*E. elatior*	Fl	1.8/1.9	6.0/7.3	6	*19.4*/9.7	4.0/5.5	0.8/0.9

*EP* edible part, *S* seeds, leaves, *A* aerial parts, *Yl* young leaves, *F* fruits, *R* rhizomes, *Ls* leaves and stems, *Ip* inner peel/cortex, *Fl* flowers, *G* grains

^*^(MoH, 2019)

Italic: Source of a certain nutrient (a 100-g portion provides 15% or more of the RDA for a certain nutrient)

Bold: High content of a certain nutrient (a 100-g portion provides 30% or more of the RDA for a certain nutrient

The average protein contents in the data obtained for these UFPs ranged from 0.02 to 18.4 g/100 g (Table 4). The contribution of their edible parts to the protein RDA varied from 0.035 to 28.3% for men and from 0.04 to 30.7% for women (Table 5).The inner peel of *Manihot esculenta* had the lowest average protein content [90, 91], while *Leucaena leucocephala* seeds had the highest [59, 92, 93]. The proteins of *L. leucocephala* seeds are fairly rich in the essential amino acids isoleucine, leucine, phenylalanine, and histidine [94]. In the study area, immature fruits that consist of seeds and pods were consumed raw or blanched as *lalapan*, in Thailand, and Central America, people also eat the young leaves, and flowers, particularly in soups [94]. Other documented UFPs that were considered sources of protein (RDA > 15%) include the leaves of *Erigeron sumatrensis* [95], *Moringa oleifera* [59, 96], and the fruits of *Piper retrofractum* [97]. Including these unconventional protein-rich food plants in their diet can diversify their protein sources, which are currently dominated by white rice and soy bean products such as tofu and tempeh. In Mexico, where the pods are eaten also raw or in soups or tacos, *L. leucocephala* is being considered as unconventional sources of protein, together with other leguminous seeds [94].

Fiber has been shown to have a positive impact on reducing the risk of various health conditions, including coronary heart disease, hypertension, diabetes, obesity, and gastrointestinal issues [98]. The average fiber contents among the documented plants varied from 0.2 to 23.6 g/100 g (Table 4), with a 100-g edible portion of these plants providing RDA percentages ranging from 0.4 to 65.6% for men and 0.5 to 78.7% for women (Table 5). Several UFPs documented in this study have been identified as notable sources of fiber, include inner peel of *Manihot esculenta* [90, 91], fruits of *Piper retrofractum* [97], *Sandoricum koetjape* [35, 99]*, Phyllanthus emblica* [100, 101], and leaves of *Gynura divaricata* [102, 103], *Crassocephalum crepidioides* [59, 104], *Moringa oleifera* [59, 96], *Ipomoea batatas* [59, 105]. Their average fiber values (ranging from 10.9 to 50.9 g/100 g) were higher than that of the commonly consumed *sawi hijau*/leaf mustard (*Brassica juncea*) whose fiber content was 2.5 g/100 g, providing 6.9–8.3% of the RDA [59, 60]. Consuming a 100-g portion of these plant parts can contribute to more than 15% of the recommended daily fiber intake for adults, thus making them valuable additions to a fiber-rich diet. Nevertheless, achieving the recommended daily intake of certain plant parts that are commonly used as spices or herbal medicine, such as *P. retrofractum* can be challenging as they are used in lower quantities compared to those consumed as main dish, or snack/delicacy.

The information on mineral content in the documented UFPs is limited, but it is indicated that the majority of them serve as good mineral resources. The average calcium (Ca) content in the obtained data ranged from 4 to 854 mg/100 g (Table 4), with a 100-g portion of edible plant parts contributing 0.4–85% of its RDA for adults (Table 5). The leaves of *Sonchus arvensis* [106, 107]*, Pilea melastomoides* [59] and *Moringa oleifera* [59, 96] represent the top two Ca contents, providing over 50% of the RDA per 100 g of the edible portion. The contributions of other interesting sources of Ca (15–30% of RDA), such as *Solanum Americanum* [52, 59, 103]*, and Solanum torvum* [59, 108, 109] berries, leaves of *Centella asiatica* [23, 110]*, Cosmos caudatus* [39, 52, 59, 111], *Crassocephalum crepidioides* [59, 104, 112, 113]*, Breynia androgyna* [52, 59, 114, 115], *Oenanthe javanica* [23, 59, 116, 117]*,* and *Ipomoea batatas* [59, 105] are comparatively higher than those of conventional vegetables in Indonesia, such as *kangkung*/water spinach (*Ipomoea aquatica*) (6.7% RDA) or *selada*/lettuce (*Lactuca sativa*) (2.2% RDA) [59, 60]. The average Fe contents of the retrieved data ranged from 0.25 to 10.5 mg/100 g (Table 4), with a 100-g portion of the edible parts providing RDA percentages of 2.8–116.7% for men and 1.4–58.3% for women (Table 5). More than half of the documented UFPs in this study were identified as iron (Fe) sources (RDA > 15%). Our recent dietary survey conducted in the study area found that the average daily Fe intake of 107 women (16.8 mg/day, Supplementary 1) falls below the RDA of 18 mg/day [60]. A deficiency in Fe can lead to the development of anemia, which is a form of malnutrition [118]. Cases of iron deficiency anemia remain prevalent in Indonesia, particularly, among women in rural area [119]. Therefore, consuming Fe-rich UFPs (RDA > 30%) can be encouraged as a dietary choice. These include edible parts of *Canna indica* [59, 120], *Acmella ciliata* [59, 121], *Bidens pilosa* [122, 123], *S. americanum* [52, 59, 103], *S. arvensis* [106, 107], *Emilia sonchifolia* [59, 103, 124], *Moringa oleifera* [59, 96],* Gynura divaricata* [102, 103], *Breynia androgyna* [52, 59, 114, 115], and *Eryngium foetidum* [103, 117, 125, 126]. These Fe-rich UFPs contain concentrations ranging from 3.5 to 10.5 mg/100 g (Table 4). However, achieving the recommended intake would require the consumption of 50–100 g (fresh weight) of their edible parts*.* The average Zn content among the retrieved data varied from 0.02 to 8.3 mg/100 g (Table 4). *C. asiatica* [23, 110] leaves exhibit the highest, followed by *O. javanica* [23, 59, 116, 117], *Clitoria ternatea* [127], *Leucaena leucocephala* [59, 92, 93], and *S. arvensis* [106, 107]. A 100-g edible portion of these Zn-rich UFPs contributes to 41.3–75.1% of Zn RDA for men and 56.8–103.2% for women (Table 5). This surpasses the amount of Zn provided by widely marketed broccoli (0.5 mg/100 g) with its corresponding contribution to the RDA for men/women (4.5/6.3%) [59].

In terms of vitamin C, the obtained data showed an average content ranging from 0.6 to 75 mg/100 g (Table 4). The edible parts of *Eryngium foetidum* [103, 117, 125, 126], *Breynia androgyna* [52, 59, 114, 115], *Phyllanthus emblica* [100, 101], *Sonchus arvensis* [106, 107], and *Moringa oleifera* [59, 96] exhibited the top five values. Several documented UFPs are found to be a rich source of vitamin C compared to some commonly consumed fruits and vegetables (Table 4). For example, the average vitamin C contents in the leaves of *E. foetidum* [103, 117, 125, 126]*, Anacardium occidentale* [39, 59, 111, 116], *B. androgyna* [52, 59, 114, 115], *M. oleifera* [59, 96], *Cosmos caudatus* [39, 52, 59, 111], fruits of *P. emblica* [100, 101] (58–75 mg/100 g) are greater than that of orange (*Citrus* × *sinensis*) at 49 mg/100 g [59]**.** Other plants, such as *Pilea melastomoides* [39, 59, 128], *Apoballis rupestris, Archidendron jiringa* [59], *Syzygium cumini* [59, 129], *Benincasa hispida* [130, 131], *Sicyos edulis* (leaves and stems) [59], *Mangifera odorata* [35, 59], and *Antidesma bunius* [52, 132, 133], have also been reported to possess a high content of vitamin C, providing more than 15% of the RDA of vitamin C for adults (Table 5). These plants have average values between 22 and 38 mg/100 g, comparable to pineapple (22 mg/100 g). Although there have been numerous documented UFPs that are regarded as vitamin C sources and are eaten raw*,* a few of them, such as *B. androgyna* and *M. oleifera* leaves, are usually cooked. Thus, it is crucial to acknowledge that the processing of food significantly affects this particular vitamin [134, 135].

Overall, the nutritional evaluation of the documented UFPs indicates their significant potential to meet dietary needs, as many of them offer high levels of protein, fiber, calcium, iron, zinc, and vitamin C. Local communities in the study area derive important nutrients from these plants. However, some UFPs, such as *Liquidambar excelsa*, *Rorippa indica*, and *Ficus virens*, lack scientific evidence regarding their nutritional values, and many of their nutritional attributes remain uninvestigated or undocumented. Limited information regarding nutrient composition is a well-known obstacle to the valorization of neglected and underutilized species [10, 136], highlighting the need for further research.

### UFP consumption

The respondents showed varying levels of UFP consumption, with 56% having moderate consumption (2–3 times a week), while 31% and 13% had high (every day or 4–6 times a week) and low consumption (once a week or less), respectively (Table 2). This implies that UFP use is common in the dietary practices of the study population. Similarly, in the Bogor district of West Java, 60% of Sundanese women reported consuming indigenous vegetables containing UFPs like *Pilea melastomoides* and *Acmella ciliata* at least three times a week [137].

Despite the common use of UFPs indicated by the FFQ results, our nutritional assessment found deficiencies in almost all micronutrients (except sodium) and fiber among the study population (Supplementary 1). Given the nutritional potential of some documented UFPs, their increased incorporation into local diets could be valuable in addressing these deficiencies and improving overall diet quality, as evidenced in other contexts. In Tanzania and the Philippines, traditional food plants have been shown to contribute to iron, calcium, and vitamin A intake among rural people [13, 138]. In Vietnam, most women in the study population reported obtaining dietary folate from wild vegetables [22]. Indigenous vegetables also contributed to micronutrient intake among women in the Lama Forest communities of southern Benin [139] and rural communities of Swaziland [13]. Additionally, high consumption of underutilized vegetables in other Indonesian studies was reported to have a positive association with perceived skin quality, β-carotene intake from *lalapan*, daily β-carotene intake, and blood β-carotene concentrations [137].

### Correlates of UFP consumption

The correlation between UFP consumption frequency and other sociodemographic and nutritional variables is summarized in Table 6. While the direct and indirect pathways to promoting UFP consumption have yet to be examined, our findings indicate that UFP consumption frequency is positively correlated with age (*r* = 0.240, *p* = 0.015), livestock possession (*r* = 0.260, *p* = 0.008), and UFP knowledge (*r* = 0.70, *p* = 0.000), but negatively correlated with family size (*r* = − 0.220, *p* = 0.02). No correlations (*p* > 0.05) were found between consumption and education, occupation, and expenditure. The association between age and the consumption of UFPs, including wild edible plants, is well-documented, with a trend of increased consumption among older individuals [3, 140, 141]. The negative correlation with household size may be due to the fact that, in larger families, the time and labor required for gathering UFPs might be redirected to other essential activities, such as farming, childcare, or wage labor [142]. However, these correlations were generally weak.

**Table 6:** Correlates of UFP consumption (*n* = 107)

Variables	^Y^UFP consumption frequency	*p* value
Age	0.240*	**0.015**
Number of family members	− 0.220*	**0.020**
Education completed	0.137	0.159
Source of income	− 0.111	0.257
Monthly expenditure (in k IDR)	0.087	0.371
Livestock inventory	0.260*	**0.008**
UFP knowledge	0.710**	**0.000**

Source of income category is a dummy variable with farmer set at 1 and non-farmer at 0. Education completed is treated as a continuous variable with values from 0 (none) to 4 (higher than high school). Correlations are reported using Pearson’s or Spearman’s Rho, according to variable distribution

^Y^The values represent the correlation coefficients (*r*) between each variable and UFP consumption. Strong correlation: 0.7 < *r* < 0.9; moderate correlation 0.5 < *r* < 0.7; weak correlation: *r* < 0.3. *p* value < 0.05 represented in bold indicates a significant difference. *, and **indicate significance at 0.05 and 0.01

In contrast, a strong positive correlation was found between UFP consumption and knowledge about UFPs (*r* = 0.70); those who cited more UFPs consumed them more frequently. On average, respondent recognized six UFP species, with the most knowledgeable cited 25 species. This finding aligns with the general view that ethnobotanical knowledge and uses of plants are closely related [143]. People who have higher ethnobotanical knowledge can use more plant species than people who have less ethnobotanical knowledge [3, 140], or vice versa. However, results from the few studies that differentiate between ethnobotanical knowledge and uses of plants showed that the two variables do not necessarily correlate, indicate that other factors may modify this relationship [141, 143]. Researchers argue that discrepancies between survey responses and actual plant use arise from the replacement of plants with commercial substitutes or lifestyle changes [143]. A study in the Bolivian Amazon comparing ethnobotanical knowledge and plant use in two communities with different levels of socioeconomic change showed no correlation between knowledge and use in a village closer to the market, suggesting erosion of ethnobotanical knowledge [141]. However, in a more isolated area where plant use has not changed drastically, ethnobotanical knowledge did correlate with plant use. Similarly, in this present study, the local communities of Rancakalong maintain their traditional way of life despite the influences of social transformation, including their knowledge and use of plant diversity preserved in local food cultures. The finding indicates the importance of ethnobotanical knowledge in sustaining the use of UFP in the area, thus the effort to promote greater use of UFPs in the local diet, should be hand in hand with effort to increase their knowledge. Additionally, further research is needed to examine between ethnobotanical knowledge and actual uses of plants in different settings to understand knowledge erosion.

We found no correlation between UFP consumption frequency and socioeconomic factors (education, occupation, household expenditure), which highlights a noteworthy aspect of the relative democratization of UFP use [64]. Although UFPs can have notable nutritional value, they are often overlooked [9, 144]. This combination often leads to these plants being unjustly labeled as “famine foods” or poor-people’s food and limits their recognition as coping strategies for the most impoverished segment of the population [64]. Our study did not find such a derogatory view toward UFPs. However, it may not fully represent the situation due to limitations in our study design’s ability to capture the economic diversity among respondents.

### Motivations of UFP consumption

**Fig. 2:**
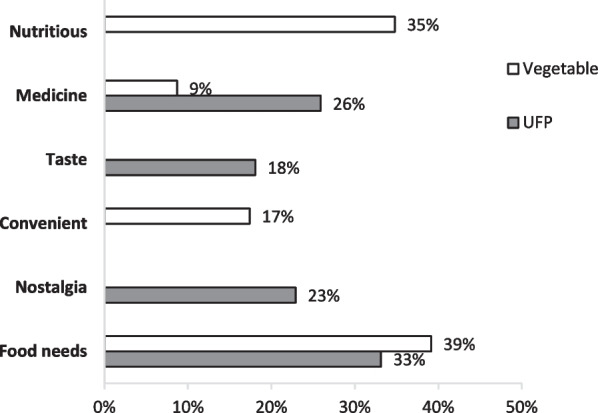
Reasons for consuming UFP versus vegetable

Given the current global decline in UFP use, research has predominantly focused on clarifying the reasons underpinning their decrease, while the motivations driving their continued consumption have garnered less attention. Recognizing these motivations can lead to more effective strategies for facilitating the necessary changes. In industrialized regions, motivations for the use of UFPs lean toward recreation and innovative food trends [61, 145], whereas traditional and indigenous communities value them for their critical role in diet, economy, and culture [146]. In the studied area, the main motivations for consuming both UFPs and vegetables were daily food needs (33% for UFPs, 39% for vegetables) (Fig. 2). There are instances where certain wild forms are occasionally brought into gardens and cultivated directly to achieve a higher yield and ensure immediate availability of food. Examples of such plants include *Cosmos caudatus, Physalis angulata Crassocephalum crepidioides,* and *Oenanthe javanica*. This practice is widely reported in rural areas of Indonesia and elsewhere, where freely accessible plants resources, including common vegetables, are used to meet daily food needs, thereby reducing household food expenses [18, 38, 147]. Additionally, two documented UFPs in this study, *Liquidambar excelsa* and *Ficus virens*, recognized as timber trees [52], were found in the forest. The locals collected their young leaves on their way to the farm field.

While economically motivated factor was perceived as necessary for both UFPs and vegetables, nutritional benefit was only recognized for vegetables (35%); none of the respondents in this study perceived UFPs as nutritionally beneficial. The findings suggest a general view of UFPs as necessary free food resources rather than as nutrient sources, indicating that the locals value UFPs for their role in the diet to ensure a reliable food supply rather than for fulfilling nutritional requirements. This highlights locals’ perception of the UFPs’ role as a key resource for food security, providing a safety net for households during times when conventional food sources might be unavailable or insufficient [148, 149]. This finding also indicates a lack of knowledge regarding UFP’s nutritional benefits, which may lead to an underestimation of these plant resources, both nutritionally and socioeconomically [148]. For example, the study site is a major sweet potato (hui Cilembu cultivar) cultivation area where the leaves are regarded as postharvest waste. Considering that sweet potato leaves is a good source of calcium (157 mg/100 g, providing 16% of the RDA for Ca), it is recommended for more consumption into include them in the locals’ diet to help alleviate Ca deficiency, particularly among women in the area (Supplementary 1). Although perceptions of sweet potato leaves may vary across cultures, there is no negative view toward sweet potato leaves (or other documented plants) in the studied villages. Instead, their low utilization stems from a lack of awareness of their valuable nutrition. Thus, raising awareness regarding nutrient-rich UFPs is necessary. In this regard, we have taken a baby step by disseminating the result of UFP nutritional values to the studied population (Table 4). We presented this information to the communities in a simple chart comparing the nutritional content of some UFP with that of conventional vegetables or fruits.

The importance of having medicinal values was more emphasized for UFPs (26% for UFPs, 9% for vegetables) (Fig. 2). This is understandable, as the use of herbal medicine is widespread in Indonesia [24, 150], and therapeutic properties are also a more pronounced motivation for consuming wild edible plants in other cultures [3, 65]. Additionally, personal factors or subjective reasons, such as taste preference (18%) and nostalgia (23%), were also more emphasized for UFPs. Alongside the traditional dietary practice of *lalapan*, individuals have developed affective (emotional) relationships with UFPs, linking consumption with nostalgic feelings often tied to cultural traditions. Some respondents described how eating *humut* (coconut heart) or boiled *cariwuh* (*Apoballis rupertis*) brought back vivid memories of their childhood or traditional ceremonies. These plants are used to prepare a ceremonial dish called *pahinum*, which is served during the celebration of a newborn’s 40th day in the study area. Behavior studies have demonstrated that food consumption is closely tied to memory [151]. The taste of UFPs as the main reason for their consumption has also been observed in other countries [152–154]. In general, local communities in Rancakalong use the diversity of food plants in preparing various dishes necessary for their ritual ceremonies [46, 48]. Overall, our findings on the motivation for UFP consumption corroborate that local communities value these underutilized resources for their critical role in diet, medicine [3, 65] and culture [146].

Despite the continued use of UFPs, most of the respondents (92%) have perceived a decline in the consumption of these plants compared to the past. Several reason were cited as probable cause of this decline: perceived reduced availability (48%), a lack of knowledge regarding UFP (30%), time constraints and convenience (13%), and a preference for improved crop varieties (9%). Reduced availability is a well-known factor associated with declining consumption of unconventional food resources, as reported in other regions in West Java, Indonesia [28], West Sumatra, Indonesia [18], and Gujarat, India [44]. It is important to note that in this present study, the perceived reduction in availability of UFPs is based on respondents’ observations; no direct measurement was conducted. Following perceived reduced availability, lack of knowledge (30%) was cited as one reason for consumption decline. This finding further supports our observation of a strong positive correlation between UFP-related knowledge and consumption (Table 2). Additionally, the diversity of plants and their associated knowledge may be closely related [3]; individuals with greater knowledge are more likely to be aware of the availability of these plants. In the study area, such individuals either cultivated the plants or took care of their natural habitats or unmanaged locations where they were typically found. For example, one respondent cultivated cariwuh (*Apoballis rupertis*) in her home garden from a plant she collected from the forest where it is typically found. While it is commonly reported that a shift in food preferences toward a greater variety has resulted in the neglect of wild or native species by local communities [3, 71], this does not entirely apply to the community in this study. Only 9% of respondents expressed a preference for improved crop varieties as one of possible reasons for declining UFP consumption. This may be because local food culture continues to play a prominent role, as evidenced by the ongoing tradition of *lalapan* consumption in their diet and the use of UFP in Rancakalong cultural ceremonies. Previous Indonesian studies also revealed that Sundanese communities residing in the Eastern Priangan areas and indigenous communities of Mentawai still highly value and prefer their traditional foods, thus sustaining the use of UFPs [37, 38].

### Implications for study area

The results of this study provide significant insights into the potential role of unconventional food plants in food and nutrition security, environmental sustainability, cultural heritage, and public health in the study area. The documentation of UFPs highlights the diversity of underutilized food plants in the area. Although the number of UFPs is fewer than in some other regions, this presents an opportunity to explore and document additional local food resources, which could enhance the role of UFPs in the local food system. This is particularly important given that local communities value these underutilized resources as a safety net when conventional foods are insufficient, thereby strengthening food security in the area.

The high variability in nutritional content, including proteins, fibers, and minerals such as calcium, iron, and zinc, further emphasizes the potential of UFPs to contribute significantly to nutritional adequacy. For example, *Leucaena leucocephala* seeds and *Moringa oleifera* leaves are rich in protein and calcium, while *Sonchus arvensis* and *Eryngium foetidum* provide ample iron and vitamin C. While the continued use of UFPs found in this study may imply that they provide such nutritional benefits to local communities, the deficiencies in almost all micronutrients and fiber observed among the study population (Supplementary 1) suggest that current consumption is inadequate. Malnutrition, which includes anemia and stunting, remains a persistent public health issue in rural areas in Indonesia [42, 43] including this study area [49]. Thus, promoting greater incorporation of nutrient-rich UFPs into the local diet would improve the nutrition of local communities and address these issues rooted in nutrient deficiencies. However, to effectively leverage UFPs for these purposes, more research on their nutritional composition is essential. Currently, some UFPs lack scientific evidence regarding their nutritional attributes, and many of these attributes remain uninvestigated or undocumented.

Furthermore, as we found that the consumption of UFPs is closely associated with knowledge about them, it is imperative that efforts to promote their greater consumption are accompanied by initiatives to raise awareness about UFPs, particularly regarding their nutritional value, which is not well recognized in the area. We also found that knowledge may be interrelated with reduced availability as perceived by the respondents. Thus, raising awareness of UFPs also has implications for preserving their biodiversity in the area. Key entry points for such efforts can include government nutritional programs aimed at promoting the diversity of local food plants (*tanaman pangan lokal*) consumption at the community level, such as the *Pangan Harapan* (Desirable Diet Pattern) [155], and *Isi Piringku* (In My Plate) [156]. Regarding the perceived declining availability of UFPs in the area, promoting their use in the local food system must go hand in hand with promoting their sustainable use. While overharvesting was not cited as the primary reason for the decreasing consumption trend in the study area, it is noteworthy that overharvesting, or using entire plants or roots, should be avoided to preserve these valuable resources [157]. At the community level, we argue that the sustainable use and conservation of these underutilized plants should be integrated into the social-ecological system in which the species are observed. Maintaining the integrity of this local ecosystem is equally important and has been partially addressed by the West Java Government through programs like the *Buruan Sae* and *Teras Hijau* (Green Terrace) [158], which promote the use of home gardens as a source of local food plants. However, UFPs were not explicitly recognized. Incorporating UFPs into such programs would raise awareness among local communities [18] and encourage cultivation, thus ensuring that households have access to nutritious food and contributing to promoting food security and nutrition in the area. Finally, creating awareness and promoting the sustainable use of UFPs can help maintain and enrich cultural heritage, as these plants are often integral to traditional practices, culinary traditions, and local identities.

The first limitation of this study is the use of *ex situ* interviews—free listing and FFQ (dietary recall)—in documenting the plants, without being accompanied by in situ survey. The in situ “walk-in-the-woods” method resulted in more detailed information on plant knowledge and use frequency than the *ex situ* interviews [75]. This is because the in situ method allows for assessing plant knowledge within its ecological context. Walking through the natural environment enables the encounter of many species that do not readily come to mind during off-forest interviews. While free listing and dietary recalls can provide quantitative data and a more general overview, such methods cannot capture the full diversity of plants known and used as effectively as the “walk-in-the-woods” survey. Consequently, the plants documented in this study are fewer compared to some other areas and lack accuracy in terms of the status of the plants “wild or cultivated”. The second limitation relates to the use of the FFQ method in measuring UFP consumption. Using the FFQ method, we cannot obtain real-time data on the contribution and accurate average amount of UFP intake in the diet, as can be measured by other dietary recalls such as the 24-h dietary recall. However, the FFQ accounts for consumption variations over a longer timeframe, capturing consumption patterns of plants that are not part of the daily diet [139]. In follow-up comparative studies, both the FFQ and 24-h recall methods will be employed to obtain comprehensive data on the contribution of UFP consumption to nutritional intake. The third limitation of this study involves certain nutrient values in Table 4, which are single data points rather than averages due to limited references. This underscores the need for careful consideration when interpreting data. Finally, although the survey was limited to only three villages in a rural site, we believe that the results sufficiently represent rural communities’ practice regarding UFPs in West Java but may not be necessarily generalizable to other populations in Indonesia. Thus, replicating this research based on a case study in other regions is advisable to elucidate more comprehensive information on UFPs’ potential contribution to improved nutrition.

## Conclusion

We documented 52 unconventional food plants commonly consumed in the diet of rural communities in the Rancakalong district of Sumedang Regency, West Java. This study not only highlights the existing diversity of underutilized plant resources in the area but also shows that the nutritional evaluation of these UFPs reveals their significant potential to meet dietary needs, as many are rich in protein, fiber, calcium, iron, zinc, and vitamin C. Despite the common use of UFPs in the local diet, the nutritional potential of some documented UFPs suggests that their greater incorporation could be valuable for addressing nutrient deficiencies and improving overall diet quality. Findings on motivation for consuming UFPs show that local communities value UFPs primarily for their critical roles in diet, medicine, and cultural traditions. While UFPs are seen as a vital resource for ensuring food security, especially in times of scarcity, there is a significant lack of awareness regarding their nutritional benefits. This knowledge gap may lead to underestimating their potential as nutritional resources. Our findings reveal a strong correlation between UFP knowledge and consumption, highlighting the need for an integrated approach to promote UFPs. Therefore, efforts to increase UFP consumption should include educational initiatives to create awareness about them and reinforce their cultural and traditional significance.

This study underlines significant insights into the potential role of UFPs in strengthening food security and improving nutrition, which can help address malnutrition issues, including anemia and stunting, in the area. Thus, the perceived declining consumption and availability of these underutilized resources represent a lost opportunity. Consequently, promoting the greater inclusion of these plants in diets and their awareness among communities must also be accompanied by advocating their sustainable use. Further, it is recommended to continue investigating poorly known nutrients of widely used species to fully enhance their contribution.

### Supplementary Information

Supplementary Material 1.

## Data Availability

A structured, organized version of the data and the voucher numbers of the voucher specimens will be available from the first author upon reasonable request.

## Abbreviations

UFP: Unconventional Food Plants
RDA: Recommended Dietary Allowances
USDA: United States Department of Agriculture
AKG: Angka Kecukupan Gizi (RDA in Indonesia)
IDR: Indonesian Rupiah (currency)

## References

[1] Nolan JM, Turner NJ. Ethnobotany: the study of people–plant relationships. In: Anderson EN, Pearsall D, Hunn E, Turner N, editors. Ethnobiology. London: Wiley-Blackwell; 2011.

[2] Leal ML, Alves RP, Hanazaki N. Knowledge, use, and disuse of unconventional food plants. J Ethnobiol Ethnomed. 2018;14:6. 29343263 10.1186/s13002-018-0209-8 PMC5773074

[3] Yangdon P, Araki T, Rahayu YYS, Norbu K. Ethnobotanical study of wild edible fruits in eastern Bhutan. J Ethnobiol Ethnomed. 2022;18:27. 35354474 10.1186/s13002-022-00526-8 PMC8966357

[4] Rahayu YYS, Araki T, Rosleine D. Factors affecting the use of herbal medicines in the universal health coverage system in Indonesia. J Ethnopharmacol. 2020;260:e112974. 10.1016/j.jep.2020.112974 32428656

[5] Khadka D, Paudel HR, Luo B, Ding M, Basnet N, Bhatta S, et al. Edible oil-producing plants in the Sinja Valley, Jumla, Nepal. Front Sustain Food Syst. 2023;7:1276988.

[6] Fongnzossie EF, Fernande C, Nyangono B, Biwole AB, Nee P, Ebai B, et al. Wild edible plants and mushrooms of the Bamenda Highlands in Cameroon: ethnobotanical assessment and potentials for enhancing food security. J Ethnobiol Ethnomed. 2020;16A:1–10. 10.1186/s13002-020-00362-8 PMC7055030 32131859

[7] Rapoport EH, Drausal BS. Edible plants. In: Levin SA, editor. Encyclopaedia of biodiversity. 2nd ed. New York: Academic Press; 2013. p. 127–32.

[8] Prescott-Allen R, Prescott-Allen C. How many plants feed the world? Conserv Biol. 1990;4:365–74.

[9] Padulosi S, Thompson J, Rudebjer P. Fighting poverty, hunger and malnutrition with neglected and underutilized species (NUS): needs, challenges and the way forward. Rome: Bioversity International; 2013.

[10] Hunter D, Borelli T, Beltrame DMO, Oliveira CNS, Coradin L, Wasike VW, et al. The potential of neglected and underutilized species for improving diets and nutrition. Planta. 2019;250:709–29. 31025196 10.1007/s00425-019-03169-4

[11] Kuhnlein HV. Food system sustainability for health and well-being of Indigenous Peoples. Public Health Nutr. 2015;18:2415–24. 25522932 10.1017/S1368980014002961 PMC10271446

[12] Popkin BM, Adair LS, Ng SW. Global nutrition transition and the pandemic of obesity in developing countries. Nutr Rev. 2012;70:3–21. 22221213 10.1111/j.1753-4887.2011.00456.x PMC3257829

[13] Powell B, Thilsted SH, Ickowitz A, Termote C, Sunderland T, Herforth A. Improving diets with wild and cultivated biodiversity from across the landscape. Food Secur. 2015;7:535–54.

[14] Flyman MV, Afolayan AJ. The suitability of wild vegetables for alleviating human dietary deficiencies. S Afr J Bot. 2006;72:492–7.

[15] FAO, IFAD, UNICEF, WFP, WHO. The state of food security and nutrition in the world 2020. Transforming food systems for affordable healthy diets Rome: FAO; 2020.

[16] Sakai Y, Rahayu YYS, Araki T. Nutritional value of canteen menus and dietary habits and intakes of university students in Indonesia. Nutrients. 2022;14:1911. 35565878 10.3390/nu14091911 PMC9105855

[17] Khoury CK, Bjorkman AD, Dempewolf H, Ramirez-Villegas J, Guarino L, Jarvis A, et al. Increasing homogeneity in global food supplies and the implications for food security. Proc Natl Acad Sci U S A. 2014;111:4001–6. 24591623 10.1073/pnas.1313490111 PMC3964121

[18] Pawera L, Khomsan A, Zuhud EAM, Hunter D, Ickowitz A, Polesny Z. Wild food plants and trends in their use: from knowledge and perceptions to drivers of change in West Sumatra, Indonesia. Foods. 2020;9:e1240. 10.3390/foods9091240 PMC7555794 32899857

[19] FAO, IFAD, UNICEF W and W. The State of Food Security and Nutrition in the World 2021. Transforming food systems for food security, improved nutrition and affordable healthy diets for all. FAO. Rome; 2021.

[20] Bharucha Z, Pretty J. The roles and values of wild foods in agricultural systems. Philos Trans R Soc B Biol Sci. 2010;365:2913–26. 10.1098/rstb.2010.0123 PMC2935111 20713393

[21] Ahmad K, Pieroni A. Folk knowledge of wild food plants among the tribal communities of Thakht-e-Sulaiman Hills, North-West Pakistan. J Ethnobiol Ethnomed. 2016. 10.1186/s13002-016-0090-2. 27059025 10.1186/s13002-016-0090-2 PMC4826518

[22] Ogle BM, Johansson M, Tuyet HT, Johannesson L. Evaluation of the significance of dietary folate from wild vegetables in Vietnam. Asia Pac J Clin Nutr. 2001;10:216–21. 11708312 10.1046/j.1440-6047.2001.00261.x

[23] Punchay K, Inta A, Tiansawat P, Balslev H, Wangpakapattanawong P. Nutrient and mineral compositions of wild leafy vegetables of the Karen and Lawa communities in Thailand. Foods. 2020;9:1–15. 10.3390/foods9121748 PMC7759793 33256047

[24] Rahayu YYS, Araki T, Rosleine D, Purwaningtyas RM. General practitioners’ attitudes toward traditional Indonesian herbal medicine and integrative care in the Universal Health Coverage System. Glob J Health Sci. 2022;14:82.

[25] Smith E, Ahmed S, Dupuis V, Running Crane M, Eggers M, Pierre M, et al. Contribution of wild foods to diet, food security, and cultural values amidst climate change. J Agric Food Syst Community Dev. 2019;9:1–24.

[26] WFO. WFO Plant List. https://wfoplantlist.org/. Accessed 1 Feb 2023.

[27] Sormin BH. Conserving biological diversity in managed tropical forests. In: Blockhus J, Dillenbeck M, Sayer J, Wegge P, editors. IUCN general assembly. Perth: IUCN; 1990.

[28] Pratama MF, Dwiartama A, Rosleine D, Abdulharis R. Documentation of Underutilized Fruit Trees (UFTs) across indigenous communities in West Java, Indonesia. Biodiversitas. 2019;20:2603–11.

[29] Walujo EB. Keanekaragaman hayati untuk pangan. Jakarta: LIPI; 2011.

[30] Santosa E, Prawati U, Sobir Mine Y, Sugiyama N. Agronomy, utilization and economics of indigenous vegetables in West Java, Indonesia. J Hortik Indones. 2015;6:125.

[31] Afrianto WF, Hasanah LN, Metananda AA. Diversity of edible plants traded in the East Jakarta traditional markets, Indonesia. Biodiversitas. 2023;24:6953–68.

[32] Purwayantie S, Suryadi UE. Plant diversity and nutrient substances of native edible plant: case study in Suka Maju and Tamao Villages, Kapuas Hulu District, West Kalimantan, Indonesia. Biodiversitas. 2020;21:842–52.

[33] Sujarwo W, Arinasa IBK, Caneva G, Guarrera PM. Traditional knowledge of wild and semi-wild edible plants used in Bali (Indonesia) to maintain biological and cultural diversity. Plant Biosyst Int J Deal All Asp Plant Biol. 2015;150:971–6.

[34] Umartani LA. Ethnobotany of community food plants on the slopes of Merapi Merbabu, Central Java, Indonesia. Int Conf Sci Eng. 2021;4:56–63.

[35] Suwardi AB, Samudra U, Navia ZI, Samudra U, Harmawan T, Samudra U, et al. Importance and local conservation of wild edible fruit plants in the East Aceh Region, Indonesia. J Conserv Sci. 2022;13:221–32.

[36] Suwardi AB, Syamsuardi S, Mukhtar E, Nurainas N. The diversity and traditional knowledge of wild edible fruits in Bengkulu, Indonesia. Ethnobot Res Appl. 2023;25:1–17.

[37] Lee SM, Nichols JD, Lloyd D, Sagari S, Sagulu F, Siregar IZ, et al. The indigenous uses of plants from Siberut, Mentawai, Indonesia. Ethnobot Res Appl. 2021;22:1–33.

[38] Hernawati D, Putra RR, Meylani V. Indigenous vegetables consumed as lalapan by Sundanese ethnic group in West Java, Indonesia: potential, traditions, local knowledge, and it’s future. S Afr J Bot. 2022;151:133–45.

[39] Andarwulan N, Kurniasih D, Apriady RA, Rahmat H, Roto AV, Bolling BW. Polyphenols, carotenoids, and ascorbic acid in underutilized medicinal vegetables. J Funct Foods. 2012;4:339–47.

[40] Roosita K, Kusharto CM, Sekiyama M, Fachrurozi Y, Ohtsuka R. Medicinal plants used by the villagers of a Sundanese community in West Java, Indonesia. J Ethnopharmacol. 2008;115:72–81. 17961941 10.1016/j.jep.2007.09.010

[41] Zumsteg IS, Weckerle CS. Bakera, a herbal steam bath for postnatal care in Minahasa (Indonesia): documentation of the plants used and assessment of the method. J Ethnopharmacol. 2007;111:641–50. 17293070 10.1016/j.jep.2007.01.016

[42] Dinkes Jabar. Profil Kesehatan Indonesia Jawa Barat tahun 2019. Bandung: Dinkes Jabar; 2019.

[43] Kemenkes RI & BPS. Laporan Pelaksanaan Integrasi Susenas Maret 2019 dan SSGBI Tahun 2019. Jakarta: BPS; 2019.

[44] Chauhan SH, Yadav S, Takahashi T, Łuczaj Ł, D’Cruz L, Okada K. Consumption patterns of wild edibles by the Vasavas: a case study from Gujarat, India. J Ethnobiol Ethnomed. 2018;14:1–20. 30157859 10.1186/s13002-018-0254-3 PMC6116503

[45] Rinandio DS, Helmanto H, Zulkarnaen RN, Primananda E. Endemic plants of Java Island, Indonesia: a dataset. Biodivers Data J. 2022;10:e84303. 36761504 10.3897/BDJ.10.e84303 PMC9848522

[46] Iskandar J, Iskandar BS. The Sundanese traditional ecological calendar and socio-cultural changes: case study from Rancakalong Java, Indonesia. In: Franco FM, Knudsen M, Hassan NH, editors. Case stud biocultural divers from Southeast Asia. Singapore: Springer; 2022. p. 79–104.

[47] Rohmatullayaly EN, Suryana, Irawan B, Iskandar J. Eksplorasi dan Sosialisasi Potensi Pangan Lokal Untuk Mendukung Kesehatan Masyarakat Di Desa Rancakalong, Kabupaten Sumedang. J Apl Ipteks untuk Masy. 2022;11:349–60.

[48] Islamiati Y, Nisa NK, Anugrah R, Rosma T, Cahyanto T. Kajian Etnobotani Budaya Ngalaksa Di Kecamatan Rancakalong Kabupaten Sumedang. J Ilm Ilmu Dasar dan Lingkung Hidup. 2020;20:24–30.

[49] Dinkes Sumedang. Profil Kesehatan Kabupaten Sumedang Tahun 2019. 2020.

[50] BPS. Kecamatan Rancakalong Dalam Angka. 2023.

[51] Silva HCH, Caraciolo RLF, Marangon LC, Ramos MA, Santos LL, Albuquerque UP. Evaluating different methods used in ethnobotanical and ecological studies to record plant biodiversity. J Ethnobiol Ethnomed. 2014;10:1–11. 24916833 10.1186/1746-4269-10-48 PMC4151377

[52] PROSEA. Plant Resources of South-East Asia. https://prosea.prota4u.org/. Accessed 11 Mar 2023.

[53] DAR. Digital Academic Repository of Naturalis Biodiversity Center. 2020; https://www.repository.naturalis.nl/. Accessed 4 Feb 2023.

[54] BHL. Biodiversity Heritage Library. 2020; https://www.biodiversitylibrary.org/. Accessed 24 Mar 2023.

[55] eFlora. Flora of China. 2020; http://www.efloras. Accessed 15 Mar 2023.

[56] Backer CA. Bakhuizen van den Brink Jr. RC. Flora of Java. Groningen: N.V.P. Noordhoff; 1963.

[57] Backer CA. Bakhuizen van den Brink Jr. RC. Flora of Java. Groningen: N.V.P. Noordhoff; 1965.

[58] Backer CA. Bakhuizen van den Brink Jr. RC. Flora of Java. Groningen: N.V.P. Noordhoff; 1968.

[59] Dirjen Kemenkes. Tabel Komposisi Pangan Indonesia (Indonesian Food Composition Table). Jakarta: Kemenkes RI; 2018.

[60] Menkes RI. Permenkes RI No. 28/2019. Jakarta: Kemenkes RI; 2019.

[61] Pinela J, Carvalho AM, Ferreira ICFR. Wild edible plants: nutritional and toxicological characteristics, retrieval strategies and importance for today’s society. Food Chem Toxicol. 2017;110:165–88. 29042290 10.1016/j.fct.2017.10.020

[62] FAO and FHI 360. Minimum dietary diversity for women: a guide for measurement. Rome: FAO; 2016.

[63] Tsunoda N, Inayama T, Hata K, Oka J. Vegetable dishes, dairy products and fruits are key items mediating adequate dietary intake for Japanese adults with spinal cord injury. Spinal Cord. 2015;53:786–90. 25962372 10.1038/sc.2015.78

[64] Deaconu A, Mercille G, Batal M. Promoting traditional foods for human and environmental health: lessons from agroecology and Indigenous communities in Ecuador. BMC Nutr. 2021;7:1–14. 33413686 10.1186/s40795-020-00395-y PMC7792355

[65] Punchay K, Inta A, Tiansawat P, Balslev H, Wangpakapattanawong P. Traditional knowledge of wild food plants of Thai Karen and Lawa (Thailand). Genet Resour Crop Evol. 2020;67:1277–99.

[66] Shin T, Fujikawa K, Moe AZ, Uchiyama H. Traditional knowledge of wild edible plants with special emphasis on medicinal uses in Southern Shan State, Myanmar. J Ethnobiol Ethnomed. 2018;14:1–13. 30016980 10.1186/s13002-018-0248-1 PMC6050729

[67] Łuczaj Ł, Lamxay V, Tongchan K, Xayphakatsa K, Phimmakong K, Radavanh S, et al. Wild food plants and fungi sold in the markets of Luang Prabang, Lao PDR. J Ethnobiol Ethnomed. 2021;17:1–27. 33499871 10.1186/s13002-020-00423-y PMC7835671

[68] Ong HG, Kim Y. The role of wild edible plants in household food security among transitioning hunter-gatherers: evidence from the Philippines. Food Secur. 2017;9:11–24.

[69] Ogle BM, Dung NNX, Do TT, Hambraeus L. The contribution of wild vegetables to micronutrient intakes among women: an example from the Mekong Delta, Vietnam. Ecol Food Nutr. 2001;40:159–84.

[70] Hoe VB. The nutritional value of indigenous fruits and vegetables in Sarawak. Asia Pac J Clin Nutr. 1999;8:24–31. 24393732 10.1046/j.1440-6047.1999.00046.x

[71] Aryal KP, Poudel S, Chaudhary RP, Chettri N, Chaudhary P, Ning W, et al. Diversity and use of wild and non-cultivated edible plants in the Western Himalaya. J Ethnobiol Ethnomed. 2018;14:1–18. 29378614 10.1186/s13002-018-0211-1 PMC5789610

[72] Kunwar RM, Mahat L, Sharma LN, Shrestha KP, Kominee H, Bussmann RW. Underutilized plant species in Far West Nepal. J Mt Sci. 2012;9:589–600.

[73] Ashagre M, Asfaw Z, Kelbessa E. Ethnobotanical study of wild edible plants in Burji District, Segan Area Zone of Southern Nations, Nationalities and Peoples Region (SNNPR), Ethiopia. J Ethnobiol Ethnomed. 2016;12:1–15. 27485265 10.1186/s13002-016-0103-1 PMC4971624

[74] Teklehaymanot T, Giday M. Ethnobotanical study of wild edible plants of Kara and Kwego semi-pastoralist people in Lower Omo River Valley, Debub Omo Zone, SNNPR; 2010. p. 2–9. 10.1186/1746-4269-6-23 PMC2933608 20712910

[75] Gallois S, Heger T, Henry AG, van Andel T. The importance of choosing appropriate methods for assessing wild food plant knowledge and use: a case study among the Baka in Cameroon. PLoS ONE. 2021;16:1–19. 10.1371/journal.pone.0247108 PMC7891729 33600479

[76] Maryanto I. Ekologi Gunung Slamet. Noerdjito I, Mas M, Partomihardjo T, editors. Purwokerto: LIPI Press; 2016.

[77] Asharo RK, Novitasari A, Azizah SDN, Saraswati RA, Setyaningsih F, Apriliani P, et al. Araceae floristic and potential study in Bogor Botanical Gardens, West Java, Indonesia. J Ris Biol Apl. 2022;4:9–18.

[78] Balitbang TOOT. Riset khusus Eksplorasi Pengetahuan Lokal Etnomedicin dan Tumbuhan Obat Berbasis Komunitas Di Indonesia (Etnis Osing Jawa Timur). 2015.

[79] Agesti RARA, Ariyanti NS, Chikmawati T, Purwanto Y. Ethnobotany of food plants used by Minangkabau Community in Lima Puluh Kota District, West Sumatra, Indonesia. Biodiversitas. 2023;24:2756–67.

[80] Septiani N, Hernawati D, Putra RR. Biodiversity of potentially “lalapan” vegetables in Kampung Adat Naga, Tasikmalaya, Indonesia. Biosfer. 2020;13:201–2015.

[81] Santhyami L, Agustina L, Agustina P. Exploring urban ethnobotany: a case study of medicinal plants traded in Gede Hardjonagoro Market, Surakarta, Indonesia. Trop J Nat Prod Res. 2024;8:6839–51.

[82] Hidayat S, Wightman G. The Medicinal Value of “lalap” (Raw Vegetable) in Sundanese Society at Bogor, West Java, Indonesia. Beagle Rec. Museums Art Gall. North. Territ. Darwin, Northern Territory Museum of Arts and Sciences, 1983; 2001. p. 7–11.

[83] Nurcahyo FD, Zen HM, Rahma HS, Triyanto A, Yasa A, Naim DM, et al. Ethnobotanical study of medicinal plants used by local communities in the Upper Bengawan Solo River, Central Java, Indonesia. Int J Bonorowo Wetl. 2024;14:25–36.

[84] Fathurrahman F, Nursanto J, Madjid A, Ramadanil R. Ethnobotanical study of “Kaili Inde” tribe in Central Sulawesi Indonesia. Emir J Food Agric. 2016;28:337–47.

[85] Gailea R, Ariffien Bratawinata A, Pitopang R, Kusuma I. The use of various plant types as medicines by local community in the enclave of the Lore-Lindu. Glob J Res Med Plants Indig Med. 2016;5:29–40.

[86] Bhagawan WS, Kusumawati D. Ethnobotanical medicinal plant study of Tengger tribe in Ranu Pani Village, Indonesia. SSRN Electron J. 2021;

[87] MoH RI. Permenkes No. 41/2014. 2014.

[88] Borelli T, Güzelsoy NA, Hunter D, Tan A, Karabak S, Uçurum HÖ, et al. Assessment of the nutritional value of selected wild food plants in Türkiye and their promotion for improved nutrition. Sustainability. 2022;14:11015.

[89] Mishra A, Swamy SL, Thakur TK, Bhat R, Bijalwan A, Kumar A. Use of wild edible plants: can they meet the dietary and nutritional needs of indigenous communities in Central India. Foods. 2021;10:e1453. 10.3390/foods10071453 PMC8307769 34201552

[90] Gani SSA, Idris S, Shamsudin R, Nor Z, Mokhtar MN. Physicochemical composition of different parts of cassava (*Manihot esculenta* Crantz) plant. Food Res. 2019;4:78–84.

[91] Sarah I, Rosnah S, Mohd Noriznan M, Mohd Zuhair MN, Siti SA. Comparative proximate composition and cyanide contents of each parts of local cassava (*Manihot esculenta* Crantz). Konvensyen Kebangs Kejuruter Pertan Dan Makanan. 2019;2019:118–22.

[92] Zapata-Campos CC, García-Martínez JE, Salinas-Chavira J, Ascacio-Valdés JA, Medina-Morales MA, Mellado M. Chemical composition and nutritional value of leaves and pods of *Leucaena leucocephala*, *Prosopis laevigata* and *Acacia farnesiana* in a xerophilous shrubland. Emir J Food Agric. 2020;32:723–30.

[93] Hernández-Santos B, Quijano-Jerónimo O, Rodríguez-Miranda J. Physical, chemical, tecno-functional, and thermal properties of *Leucaena leucocephala* seed. Food Sci Technol. 2022;42:1–9.

[94] Sethi P, Kulkarni PR. *Leucaena leucocephala* a nutrition profile. Food Nutr Bull. 1995;16:1–16.

[95] Nduche MU, Edeoga HO, Omosun G, Nwankwo D. Evaluation of the chemical composition of five Nigerian medicinal plants. IOSR J Pharm Biol Sci. 2015;10:27–31.

[96] Lim TK. *Moringa oleifera*. Edible medicinal and non-medicinal plants. Dordorecht: Springer; 2012.

[97] Jadid N, Arraniry B, Hidayati D, Purwani K, Wikanta W, Hartanti S, et al. Proximate composition, nutritional values and phytochemical screening of *Piper retrofractum* vahl. fruits. Asian Pac J Trop Biomed. 2018;7:37–43.

[98] Ljubicic M, Saric MM, Rumbak I, Baric IC, Komes D, Satalic Z, et al. Knowledge about dietary fibre and its health benefits: a cross-sectional survey of 2536 residents from across Croatia. Med Hypotheses. 2017;105:25–31. 28735648 10.1016/j.mehy.2017.06.019

[99] Wongsariya K, Kanchanadumkerng P. Proximate composition of the edible part of purple passion fruit and santol and in vitro prebiotic activity of crude polysaccharide extracts. Food Res. 2021;5:406–12.

[100] Semwal P, Painuli S, Jamloki A, Rauf A, Rahman MM, Olatunde A, et al. Himalayan wild fruits as a strong source of nutraceuticals, therapeutics, food and nutrition security. Food Rev Int. 2022;39:6500–36.

[101] Barthakur NN, Arnold NP. Chemical analysis of the emblic (*Phyllanthus emblica* L.) and its potential as a food source. Sci Hortic (Amsterdam). 1991;47:99–105.

[102] Jaiboon V, Boonyanupahap J, Suwansri S, Ratanatraiwong P, Hansawasdi C. Alpha amylase inhibition and roasting time of local vegetables and herbs prepared for diabetes risk reduction chili paste. Asian J Food Agro-Ind. 2010;3:1–12.

[103] Xu Y, Liang D, Wang GT, Wen J, Wang RJ. Nutritional and functional properties of wild food-medicine plants from the coastal region of South China. J Evid Based Integr Med. 2020;25:1–13. 10.1177/2515690X20913267 PMC7163236 32297524

[104] Arawande JO, Komolafe EA, Imokhuede B. Nutritional and phytochemical compositions of fireweed (*Crassocephalum crepidioides*). J Agric Technol. 2013;9:439–49.

[105] Sun H, Mu T, Xi L, Zhang M, Chen J. Sweet potato (*Ipomoea batatas* L.) leaves as nutritional and functional foods. Food Chem. 2014;156:380–9. 24629984 10.1016/j.foodchem.2014.01.079

[106] Filho DPXG, Barreira TF, Pinheiro-Sant’Ana HM. Chemical composition and nutritional value of three sonchus species. Int J Food Sci. 2022;2022:1–9. 10.1155/2022/4181656 PMC8913140 35282307

[107] Saha S, Saha S, Mandal SK, Rahaman CH. Unconventional but valuable phytoresources: exploring the nutritional benefits of 18 wild edible Asteraceae from West Bengal, India. Genet Resour Crop Evol. 2023;70:2161–92.

[108] Nadeeshani H, Samarasinghe G, Wimalasiri S, Silva R, Hunter D, Madhujith T. Comparative analysis of the nutritional profiles of selected Solanum species grown in Sri Lanka. J Food Compos Anal. 2021;99:e103847.

[109] Lim TK. *Solanum torvum*. Edible medicinal and non-medicinal plants. 2013. p. 429–40.

[110] Ajayi OA, Olumide MD, Tayo GO, Akintunde AO. Evaluation of chemical and elemental constituents of *Centella asiatica* leaf meal. Afr J Agric Res. 2020;16:661–6.

[111] Institute for Medical Research. Malaysian Food Composition Databased (MyFCD). MyFCD Program. https://myfcd.moh.gov.my/index.html. Accessed 22 June 2024.

[112] Ismail MA, Ng X, Yee CF. Nutritional profile and antioxidative properties of selected tropical wild vegetables. Int Food Res J. 2012;19:1487–96.

[113] Adjatin A, Dansi A, Badoussi E, Sanoussi A, Dansi M, Azokpota P, et al. Proximate, mineral and vitamin C composition of vegetable Gbolo [Crassocephalum rubens (Juss. ex Jacq.) S. Moore and *C. crepidioides* (Benth.) S. Moore] in Benin. Int J Biol Chem Sci. 2013;7:319.

[114] Petrus AJA. *Sauropus androgynus* (L.) Merrill-A potentially nutritive functional leafy-vegetable. Asian J Chem. 2013;25:9425–33.

[115] Zhang B, Cheng J, Zhang C, Bai Y, Liu W, Li W, et al. *Sauropus androgynus* L. Merr.-A phytochemical, pharmacological and toxicological review. J Ethnopharmacol. 2020;257:e112778. 10.1016/j.jep.2020.112778 32205260

[116] Kongkachuichai R, Charoensiri R, Yakoh K, Kringkasemsee A. Nutrients value and antioxidant content of indigenous vegetables from Southern Thailand. Food Chem. 2015;173:838–46. 25466097 10.1016/j.foodchem.2014.10.123

[117] Narzary H, Swargiary A, Basumatary S. Proximate and vitamin C analysis of wild edible plants consumed by Bodos of Assam, India. J Mol Pathophysiol. 2015;4:128–33.

[118] Quintaes KD, Diez-Garcia RW. The importance of minerals in the human diet. In: de la Guardia M, Garrigues S, editors. Handbook of mineral elements in food. Oxford: Wiley; 2015. p. 1–21.

[119] MoH. Hasil Utama Riskesdas 2018 (Basic Health Survey 2018). Jakarta; 2018.

[120] Lim TK. *Canna indica*. Edible medicinal and non-medicinal plants. Dordorecht: Springer; 2016. p. 69–81.

[121] Konsam SC, Devi KT, Salam JS, Singh PK. Biochemical constituents and nutritive evaluation of some less known wild edible plants from Senapati district, Manipur, India. Not Sci Biol. 2016;8:370–2.

[122] Ogle BM, Grivetti LE. Legacy of the chameleon: edible wild plants in the kingdom of Swaziland, Southern Africa. A cultural, ecological, nutritional study. Part IV-nutritional analysis and conclusions. Ecol Food Nutr. 1985;17:41–64.

[123] Oduntan AO, Fasoyiro SB, Akinfasoye JA, Adeboyejo FO, Akintoye HA. Antioxidant and proximate properties of underutilized vegetables in western Nigeria. Acta Hortic. 2018;1225:255–60.

[124] Morshed MM, Rana MS, Emran TB, Sohel MD, Kawsar MH. Nutritional analysis and mineral content determination of Emilia sonchifolia DC. Bangladesh Pharm J. 2021;24:54–60.

[125] Singh S, Singh DR, Salim KM, Srivastava A, Singh LB. Estimation of proximate composition, micronutrients and phytochemical compounds in traditional vegetables from Andaman and Nicobar Islands. Int J Food Sci Nutr. 2011;62:765–73. 21615278 10.3109/09637486.2011.585961

[126] Devi OS, Komor P. Edible bioresources & livelihoods. Guwahati: Assam State Biodiversity Board; 2016.

[127] Neda GD, Rabeta MS, Ong MT. Chemical composition and anti-proliferative properties of flowers of *Clitoria ternatea*. Int Food Res J. 2013;20:1229–34.

[128] Muliasari H, Ananto AD, Ihsan M. Analisis kandungan nutrisi buah rengga (*Amomum dealbatum* Roxb). Agrotek. 2019;6:71–6.

[129] Sehwag S, Das M. Nutritive, therapeutic and processing aspects of Jamun, *Syzygium cuminii* (L.) Skeels-An overview. Indian J Nat Prod Resour. 2014;5:295–307.

[130] Aqilah N, Zaini M, Anwar F, Abdul A, Saari N. Kundur [*Benincasa hispida* (Thunb.) Cogn.]: a potential source for valuable nutrients and functional foods. Food Res Int. 2011;44:2368–76.

[131] Lim TK. *Benincasa hispida* cv-gr. fuzzy gourd. Edible medicinal and non-medicinal plants. Dordorecht: Springer; 2012. p. 1–1088.

[132] Islary A, Sarmah J, Basumatary S. Nutritional value, phytochemicals and antioxidant properties of two wild edible fruits (*Eugenia operculata* Roxb. and *Antidesma bunius* L.) from Assam, North-East India. Med J Nutr Metab. 2017;10:29–40.

[133] Lim TK. *Antidesma bunius*. Edible medicinal and non-medicinal plants. Dordorecht: Springer; 2012. p. 220–4.

[134] Berry-Ottaway P. Stability of vitamins during food processing and storage. In: Skibsted LH, Risbo J, Andersen ML, editors. Chemical deterioration and physical instability of food and beverages. Cambridge: Woodhead Publishing Limited; 2010. p. 539–60.

[135] Listiana E, Rosmala Mustapa, Kohongia A, Parisa S, Nusi DP. The effect of processing on the degradation of vitamin C in cassava leaf vegetables (in Indonesian). Pros Semin Nas Mini Ris Mhs. 2022. p. 31–5.

[136] Grivetti LE, Ogle BM. Value of traditional foods in meeting macro- and micronutrient needs: the wild plant connection. Nutr Res Rev. 2000;13:31–46. 19087432 10.1079/095442200108728990

[137] Amrinanto AH, Hardinsyah PE. The eating culture of the Sundanese: does the traditional salad (Lalapan) improve vegetable intake and blood β-carotene concentration? Future Food J Food Agric Soc. 2019. 10.17170/kobra-20190709593. 10.17170/kobra-20190709593

[138] Borelli T, Hunter D, Powell B, Ulian T, Mattana E, Termote C, et al. Born to eat wild: an integrated conservation approach to secure wild food plants for food security and nutrition. Plants. 2020;9:1299. 33019632 10.3390/plants9101299 PMC7601573

[139] Boedecker J, Termote C, Assogbadjo AE, Van Damme P, Lachat C. Dietary contribution of wild edible plants to women’s diets in the buffer zone around the Lama forest, Benin: an underutilized potential. Food Secur. 2014;6:833–49.

[140] Cruz MP, Peroni N, Albuquerque UP. Knowledge, use and management of native wild edible plants from a seasonal dry forest (NE, Brazil). J Ethnobiol Ethnomed. 2013;9:1–10. 24279311 10.1186/1746-4269-9-79 PMC4176140

[141] Reyes-garcía V, Vadez V, Leonard W, Wilkie D. Knowledge and consumption of wild plants: a comparative study in two Tsimane’ villages in the Bolivian Amazon. Ethnobot Res Appl. 2005;3:201–7.

[142] Harris M. Good to eat: riddles of food and culture. New York: Simon and Schuster; 1985.

[143] De Albuquerque UP. Re-examining hypotheses concerning the use and knowledge of medicinal plants : a study in the Caatinga vegetation of NE Brazil. J Ethnobiol Ethnomed. 2006. 10.1186/1746-4269-2-30. 16872499 10.1186/1746-4269-2-30 PMC1557484

[144] McBurney RPH, Griffin C, Paul AA, Greenberg DC. The nutritional composition of African wild food plants: from compilation to utilization. J Food Compos Anal. 2004;17:277–89.

[145] Schunko C, Grasser S, Vogl CR. Explaining the resurgent popularity of the wild: motivations for wild plant gathering in the biosphere reserve Grosses Walsertal. Austria J Ethnobiol Ethnomed. 2015;11:e55. 10.1186/s13002-015-0032-4 PMC4488988 26122103

[146] Kuhnlein HV, Erasmus B, Spigelski D. Indigenous peoples’ food systems: the many dimensions of culture, diversity and environment for nutrition and health. Rome: Food and Agriculture Organization; 2009.

[147] Bvenura C, Afolayan AJ. The role of wild vegetables in household food security in South Africa: a review. Food Res Int. 2015;76:1001–11.

[148] Guzo S, Lulekal E, Nemomissa S. Ethnobotanical study of underutilized wild edible plants and threats to their long-term existence in Midakegn District, West Shewa Zone, Central Ethiopia. J Ethnobiol Ethnomed. 2023;19:1–19. 37452368 10.1186/s13002-023-00601-8 PMC10347739

[149] Tebkew M, Asfaw Z, Zewudie S. Underutilized wild edible plants in the Chilga District, northwestern Ethiopia: focus on wild woody plants. Agric Food Secur. 2014;3:1–16.

[150] Rahayu YYS, Araki T, Rosleine D. Predictors of the use of traditional medicines in the universal health coverage system in Indonesia. Glob J Health Sci. 2021;13:24–35.

[151] Reid CA, Green JD, Buchmaier S, McSween DK, Wildschut T, Sedikides C. Food-evoked nostalgia. Cogn Emot. 2023;37:34–48. 36331076 10.1080/02699931.2022.2142525

[152] Thakur D, Sharma A, Uniyal SK. Why they eat, what they eat: patterns of wild edible plants consumption in a tribal area of western Himalaya. J Ethnobiol Ethnomed. 2017;13:1–12. 29233181 10.1186/s13002-017-0198-z PMC5727875

[153] Somnasang P, Moreno-Black G. Knowing, gathering and eating: knowledge and attribute about wild food in an Asian village in North-eastern Thailand. J Ethnobiol. 2000;20:197–216.

[154] Sõukand R. Perceived reasons for changes in the use of wild food plants in Saaremaa, Estonia. Appetite. 2016;107:231–41. 27521164 10.1016/j.appet.2016.08.011

[155] Bapanas. Peraturan Badan Pangan Nasional RI No. 11/2023. Bapanas; 2022.

[156] MoH RI. Isi piringku. Kementeri. Kesehat. RI Direktorat Jenderal Kesehat. Masy. 2022; p. 1. https://kesmas.kemkes.go.id/konten/133/0/062511-isi-piringku. Accessed 10 Aug 2023.

[157] FairWild Foundation. FairWild guidance manual for establishing species and area management plans for low risk plant species. FairWild Resour. Assess. Weinfelden: FairWild Foundation; 2014.

[158] Purnomo D, Sitepu GL, Nugraha YR, Permana Rosiyan MB. Social metabolism in Buruan SAE: individual rift perspective on urban farming model for food independence in Bandung, Indonesia. Sustainability. 2023;15:e10273.

[159] Pradityo T, Santoso N, Zuhud EA. Etnobotany in Dayak Iban’s Tembawang Sungai Mawang Villagge, West Kalimantan. Media Konserv. 2016;21:183–98.

[160] Cita KD. Ethnobotany of food plant used by Sundanese Ethnic in Nyangkewok Hamlet, Kalaparea Village, Sukabumi District, Indonesia. Asian J Ethnobiol. 2020. 10.13057/asianjethnobiol/y030103. 10.13057/asianjethnobiol/y030103

[161] Dewi AP, Peniwidiyanti P, Hariri MR, Hutabarat PWK, Martiansyah I, Lailaty IQ, et al. Ethnobotany of food, medicinal, construction and household utilities producing plants in Cikaniki, Gunung Halimun Salak National Park, Indonesia. J Mt Sci. 2023;20:163–81.

[162] Hernawati D, Putra RR. Tumbuhan Lalapan Masyarakat Sunda. 2022.

[163] Suwardi AB, Navia ZI, Harmawan T, Syamsuardi, Mukhtar E. Ethnobotany and conservation of indigenous edible fruit plants in south Aceh, Indonesia. Biodiversitas. 2020;21:1850–60.

[164] Navia ZI, Suwardi AB, Harmawan T, Syamsuardi, Mukhtar E. The diversity and contribution of indigenous edible fruit plants to the rural community in the Gayo highlands, Indonesia. J Agric Rural Dev Trop Subtrop. 2020;121:89–98.

[165] Adnan A, Navia ZI, Jamil M, Suwardi AB. The diversity and traditional knowledge of wild edible vegetables in Aceh, Indonesia. Ethnobot Res Appl. 2023. 10.32859/era.26.49.1-16. 10.32859/era.26.49.1-16

[166] Suwardi AB, Navia ZI, Harmawan T, Mukhtar E. The diversity of wild edible fruit plants and traditional knowledge in West Aceh region, Indonesia. Artic J Med Plants Stud. 2019;285:285–90.

[167] Rahayu M, Kazuhiro H. The role of plants on the traditional life of local society in Gunung Halimun National Park. West Java Ber Biol. 2004;7:17–23.

[168] Sujarwo W, Caneva G. Using quantitative indices to evaluate the cultural importance of food and nutraceutical plants: comparative data from the Island of Bali (Indonesia). J Cult Herit. 2016;18:342–8.

[169] Syafni N, Bakhtiar A. Studi Etnobotani Penggunaan Tumbuhan Paku sebagai Obat Tradisional di Siberut Tengah, Kepulauan Mentawai. J Biol Univ Andalas. 2021;10:10–4.

[170] Rahayu M, Rustiami H. Etnobotani masyarakat samawa pulau sumbawa. Scr Biol. 2017;4:235–45.

[171] Afrianto WF, Tamnge F, Hasanah LN. Review: a relation between ethnobotany and bioprospecting of edible flower Butterfly Pea (*Clitoria ternatea*) in Indonesia. Asian J Ethnobiol. 2020;3:51–61.

[172] Umartani LA, Nahdi MS. Ethnobotanical study of edible plant communities on the slopes of Mount Merapi and Merbabu, Indonesia. Biol Med Nat Prod Chem. 2021;10:33–9.

[173] Haryanti ES, Diba F. Etnobotani tumbuhan berguna oleh masyarakat sekitar kawasan KPH model Kapuas Hulu. J Hutan Lestari. 2015;3:434–45.

[174] Sujarwo W, Keim AP, Savo V, Guarrera PM, Caneva G. Ethnobotanical study of Loloh: traditional herbal drinks from Bali (Indonesia). J Ethnopharmacol. 2015;169:34–48. 25861955 10.1016/j.jep.2015.03.079

[175] Syamsuardi, Mukhtar E, Nurainas, Suwardi AB. Diversity and use of wild edible fruits in the Bukit Rimbang-Bukit Baling Wildlife Reserve, Kampar, Riau, Indonesia. Biodiversitas. 2022;23:5035–42.

[176] Suwardi AB, Navia ZI, Harmawan T, Seprianto, Syamsuardi, Mukhtar E. Diversity of wild edible fruit plant species and their threatened status in the Aceh Province, Indonesia. Biodiversitas. 2022;23:1310–8.

[177] Maimunah M, Hayati A, Zayadi H. Studi etnobotani tumbuhan legendaris Pulau Bawean Jawa Timur. Filogeni J Mhs Biol. 2021;1:47–56.

[178] Lim TK. *Mangifera foetida*. Edible medicinal and non-medicinal plants. Dordorecht: Springer; 2012. p. 82–6.

[179] Nugraha T, Mulkiya K, Kodir RA. Antioxidant activity test on different fraction and determination of flavonoid level from Jalantir leaves (*Erigeron sumatrensis* Retz.) from West Java Indonesia (In Indonesian). Pros Farm. 2016. p. 755–62.

[180] Babarinde GO, Abioye VF, Omobitan O, Raji K. Comparative study of proximate, chemical and physicochemical properties of less explored tropical leafy vegetables. J Northeast Agric Univ (English Ed). 2018;25:16–23.

[181] Lim TK. *Lagenaria siceraria*. Edible medicinal and non-medicinal plants. Dordorecht: Springer; 2012. p. 298–313.

[182] Ho L, Bhat R. Exploring the potential nutraceutical values of durian (*Durio zibethinus* L.): an exotic tropical fruit. Food Chem. 2015;168:80–9. 25172686 10.1016/j.foodchem.2014.07.020

[183] Aziz NAA, Jalil AMM. Bioactive compounds, nutritional value, and potential health benefits of indigenous durian (*Durio zibethinus* Murr.): a review. Foods. 2019;8:e96. 10.3390/foods8030096 PMC6463093 30871187

[184] Brown MJ. Durio: a bibliographic review. New Delhi: IPGRI; 1997.

[185] Nayak J, Chand BU. Analysis of some nutritional properties in eight wild edible fruits of Odisha, India. Int J Curr Sci. 2015;14:55–62.

[186] Zannah F, Kamaliah, Pramudiyanti, Ayatusaadah, Hidayati N. Exploration of the potential of local plants of *Melastoma malabatchricum* fruit for food fortification. J Trop Life Sci. 2022;12:333–8.

[187] Turi CE, Liu Y, Ragone D, Murch SJ. Breadfruit (*Artocarpus altilis* and hybrids): a traditional crop with the potential to prevent hunger and mitigate diabetes in Oceania. Trends Food Sci Technol. 2015;45:264–72.

[188] Pereira GA, Ferreira Tomé PH, Arruda HS, Fragiorge EJ, Ribeiro PR. Caracterização físico-química e atividade antioxidante do fruto calabura (*Muntingia calabura* L.). Braz J Food Res. 2016;7:67–79.

[189] Mastuki SN, Ismail SMMFN, Saad N. Muntingia calabura: chemical composition, bioactive component and traditional uses. In: Mariod AA, editor. Wild fruits: composition, nutritional value and products. Ghibaish: Springer; 2019.

[190] Lim TK. *Muntingia calabura*. Edible medicinal and non-medicinal plants. Dordorecht: Springer; 2012. p. 486–92.

[191] Yan SW, Ramasamy R, Banu N, Alitheen M, Yan SW, Ramasamy R, et al. A comparative assessment of nutritional composition, total phenolic, total flavonoid, antioxidant capacity, and antioxidant vitamins of two types of Malaysian underutilized fruits (*Averrhoa bilimbi* and *Averrhoa carambola*). Int J Food Prop. 2013;16:1231–44.

[192] Kumar KA, Gousia S, Anupama M, Latha JNL. A review on phytochemical constituents and biological assays of *Averrhoa bilimbi*. Int J Pharm Pharm Sci Res. 2013;3:136–9.

[193] Ferreira JN, Pinheiro-Sant’Ana HM, Lucia CMD, Teixeira RDBL, Cardoso LM. Chemical composition, vitamins, and minerals of family farming biribiri (*Averrhoa bilimbi* L.) in the Middle Doce River region, Minas Gerais, Brazil. Food Technol. 2022;52:1–9.

[194] Lim TK. *Averrhoa bilimbi.* Edible medicinal and non-medicinal plants. Dordrecht: Springer; 2011. p. 448–53.

[195] Lim TK. *Phyllanthus acidus*. Edible medicinal and non-medicinal plants. Dordrecht: Springer; 2012. p. 252–7.

[196] Leterme P, Buldgen A, Estrada F, Londoño AM. Mineral content of tropical fruits and unconventional foods of the Andes and the rain forest of Colombia. Food Chem. 2006;95:644–52.

[197] Brooks R, Goldson-barnaby A, Bailey D. Nutritional and medicinal properties of phyllanthus. Int J Fruit Sci. 2020;20:1706–10.

[198] Iwansyah AC, Surahman DN, Hidayat DD, Luthfiyanti R, Indriati A, Ardiansyah CE. Comparative evaluation of proximate composition and vitamin C of *Physalis angulata* Linn and *Physalis peruviana* Linn in West Java, Indonesia. IOP Conf Ser Earth Environ Sci. 2020;462:e012012.

[199] Azeez SO, Faluyi JO. Proximate analysis, Vitamin C, anti-nutrients and mineral composition of four Nigerian species of Physalis and *Solanum nigrum*. Acta Hortic. 2019;1238:81–91.

[200] Aliero AA, Usman H. Leaves of ground cherry (*Physalis angulata* L.) may be suitable in alleviating micronutrient deficiency. Food Sci Technol. 2016;4:89–94.

[201] Lim TK. *Physalis angulate*. Edible medicinal and non-medicinal plants. Dordorecht: Springer; 2013. p. 283–99.

[202] Lim TK. *Etlingera elatior*. Edible medicinal and non-medicinal plants. Dordorecht: Springer; 2014. p. 834–43.

[203] Jeevani Osadee Wijekoon MM, Karimand AA, Bhat R. Evaluation of nutritional quality of torch ginger (*Etlingera elatior* Jack.) inflorescence. Int Food Res J. 2011;18:1415–20.

