# Supplementary material for: Exploring unconventional food plants used by local communities in a rural area of West Java, Indonesia: ethnobotanical assessment, use trends, and potential for improved nutrition
Source: J Ethnobiol Ethnomed. 2024 Jul 19;20:68. doi: 10.1186/s13002-024-00710-y (PMC11264525; doi:10.1186/s13002-024-00710-y)
Supplement: Supplementary file 1 — Supplementary Material 1. [file 13002_2024_710_MOESM1_ESM.docx]

Supplementary 1

Primary data on dietary nutrient intake of 107 women in this study.

| Table Supplementary 1. The mean of daily nutrient intake of women in the study (n=107) and the percentage of those who met the Recommended Daily Allowance/RDA | | | | | | |  |
| --- | --- | --- | --- | --- | --- | --- | --- |
|  |  |  |  |  |  |  |  |
| Nutrients | | Mean ± SD | | | Recommended intake^*^ | Women met the RDA (%) |  |
| Energy (kcal) | | 1935.5 | ± | 542.1 | 2013 | 43.0 |  |
| Protein (g) | | 62.2 | ± | 20.1 | 61 | 52.3 |  |
| Fat (g) | | 63.2 | ± | 22.9 | 58 | 60.7 |  |
| Carbohydrate (g) | | 287.4 | ± | 89.4 | 302 | 39.3 |  |
| Fiber (g) | | 10.5 | ± | 5.0 | 28 | 0.9 |  |
| Calcium (mg) | | 683.6 | ± | 280.0 | 1080 | 9.3 |  |
| Phosphorus (mg) | | 933.2 | ± | 309.6 | 810 | 76.6 |  |
| Iron (mg) | | 16.8 | ± | 12.5 | 13 | 62.6 |  |
| Sodium (mg) | | 3101.3 | ± | 1689.6 | 1440 | 92.5 |  |
| Potassium (mg) | | 1712.9 | ± | 640.4 | 4760 | 0 |  |
| Zinc (mg) | | 9.6 | ± | 3.8 | 8 | 64.5 |  |
| Vitamin A (µg) | | 433.4 | ± | 818.4 | 600 | 10.3 |  |
| Vitamin C (mg) | | 69.0 | ± | 69.7 | 75 | 28.0 |  |
| ^*^Averaged RDA value for Indonesia women 16-80 years (Permenkes RI No. 28/2019). | | | | | | |  |
|  |  |  |  |  |  |  |  |

Quantitative estimates of respondent's nutrient intake were obtained through a 24-hour dietary recall method repeated over three consecutive days. Respondents listed all food items consumed in the previous 24 hours, including details on ingredients in mixed dishes, portion sizes, and preparation methods [1][2]. Each ingredient was converted into the 13 nutrient contents and subsequently calculated in reference to the food composition table [3]. The design of questionnaires, data collection, and analysis related to the nutritional variables were conducted under the supervision of a nutritionist.

**References**

1. Deaconu A, Mercille G, Batal M. Promoting traditional foods for human and environmental health : lessons from agroecology and Indigenous communities in Ecuador. BMC Nutr. 2021;7:1–14.

2. Sakai Y, Rahayu YYS, Araki T. Nutritional Value of Canteen Menus and Dietary Habits and Intakes of University Students in Indonesia. Nutrients. 2022;14:1911.

3. Dirjen Kemenkes. Tabel Komposisi Pangan Indonesia (Indonesian Food Composition Table). Jakarta: Kemenkes RI; 2018.
